# Hierarchical Membrane for Photoelectrostatically Gated Monovalent‐Divalent Ion Separation

**DOI:** 10.1002/anie.7341167

**Published:** 2026-09-10

**Authors:** Enis Oğuzhan Eren, Xuesong Xie, Zhi Li, Paolo Giusto

**Affiliations:** ^1^ Department of Colloid Chemistry Max Planck Institute of Colloids and Interfaces Potsdam Germany; ^2^ Department of Chemical and Materials Engineering University of Alberta Edmonton Alberta Canada

**Keywords:** carbon nitride, electrodialysis, ionic liquids, membranes, monovalent‐divalent

## Abstract

Monovalent‐divalent ion separation remains a significant challenge for the selective recovery of ions from aqueous solutions. Here, we report a hierarchical membrane composed of a poly(ionic liquid) photo‐grafted onto a carbon nitride thin film supported on porous anodic aluminum oxide. Under concentration gradient‐driven natural diffusion, the membrane establishes a baseline level of monovalent‐divalent selectivity. However, upon application of light and bias as external stimuli, the hierarchical membrane enables light‐assisted modulation of ion transport, resulting in a significant increase in monovalent‐divalent selectivity by over 200 times for Li^+^/Mg^2+^ and over 500 times for K^+^/Mg^2+^. Ion selectivity arises from hydration‐dependent mobility and electrostatic confinement within charged nanochannels. This work establishes a simple method to prepare hierarchical membranes for dynamically regulating ion transport and provides design principles for advanced monovalent‐divalent ion separation.

## Graphical abstract

We report a hierarchical poly(ionic liquid)–carbon nitride membrane supported on anodic alumina for efficient monovalent/divalent ion separation. Confined nanochannels and tailored interfacial ion interactions enable exceptional K^+^/Mg^2^^+^ ( > 500) and Li^+^/Mg^2^^+^ ( > 200) selectivities, offering a promising strategy for advanced aqueous ion separations.

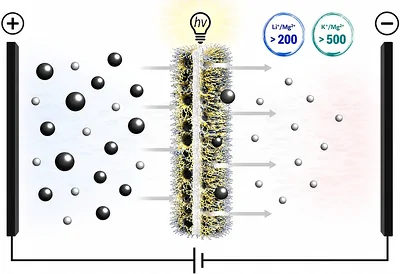

## 1 Introduction

Selective control of ionic transport in confined nanochannels plays a fundamental role in membrane science, energy storage, and biological regulation [1, 2, 3, 4]. In synthetic membranes, ion selectivity is typically governed by fixed structural parameters such as pore size, surface charge density, and chemical functionality. While these static design principles enable size‐ and charge‐based discrimination, they limit the ability to dynamically program transport pathways under operating conditions. In contrast, biological ion channels achieve adaptive selectivity through stimuli‐responsive modulation of local electrostatic environments, enabling directional transport and gating in nonequilibrium conditions [5, 6]. Translating such dynamic electrostatic control into robust synthetic nanochannels in confined spaces is challenging. At the nanoscale, ion transport is governed by the polarizability and electrostatic interaction of the membrane with the ionic medium and the ion's physicochemical properties, such as hydration energy and size [7, 8, 9]. The combined influence of these factors becomes particularly difficult in complex multi‐ion environments.

A key example is lithium extraction from natural brines, which represents both a technically complex and industrially relevant case. Lithium is essential for rechargeable battery technologies driving the global energy transition and is classified as a critical raw material due to its low abundance and uneven geographic distribution [10, 11]. Although brines are the most abundant lithium source [11, 12], conventional extraction relies on prolonged evaporation and multiple precipitation steps to remove coexisting ions [13], generating substantial chemical waste and raising sustainability concerns [14, 15]. In this context, the main challenge lies in separating Li^+^ from Mg^2+^; despite its larger hydrated radius, Mg^2+^ has an ionic radius similar to that of Li^+^, making recovery difficult under conditions involving partial dehydration [16, 17, 18]. While Na^+^, K^+^, and Ca^2+^ can generally be removed more readily [19], Mg^2+^ remains the primary bottleneck in lithium recovery [20, 21, 22, 23]. Among the most promising membrane‐based approaches to address this challenge to date are nanofiltration and electrodialysis [22, 24, 25, 26, 27]. Nanofiltration is a pressure‐driven process in which ion separation arises from pore size and surface charge interactions, whereas electrodialysis drives ion transport through alternating cation‐ and anion‐exchange membranes under an applied electric field. In both cases, separation is partially governed by Donnan exclusion, where charged membranes preferentially reject multivalent ions such as Mg^2+^ over Li^+^; however, strong electrostatic exclusion can also hinder Li^+^ transport, limiting selectivity‐recovery trade‐offs [20]. Moreover, these approaches rely primarily on static membrane properties, providing limited control over interfacial charge during operation, and combining pressure‐ and field‐driven transport concepts could, in principle, enhance performance while reducing energy demand [28, 29]. Biological membranes, for example, operate in complex environments, where confined and charged nanochannels regulate ion transport through coupled size exclusion, hydration effects, and electrostatic interactions [30, 31]. A defining feature of biological ion channels is their ability to dynamically regulate transport through stimulus‐responsive gating, enabling ion discrimination under nonequilibrium conditions [5, 32].

Inspired by biological ionic channels, we designed a hierarchical membrane comprising poly(ionic liquid) (PIL) and a photoactive carbon nitride (CN) interlayer that dynamically modulates ion transport. The membrane consists of a three‐layer architecture, in which the PIL layer is photografted from a CN thin film deposited onto a porous anodic aluminum oxide (AAO) support [33, 34]. The PIL layer provides ion selectivity through its high charge density and electrostatic interactions, favoring monovalent ion transport. Under illumination, the CN interlayer generates surface charges that dynamically modulate the electrostatic boundary conditions within the confined nanochannels [35, 36]. We define this stimulus‐responsive modulation of ionic transport as photoelectrostatic gating, whereby surface charges induce an electrostatic modulation of the ion migration pathways and amplify monovalent‐divalent selectivity. As a result, monovalent‐divalent selectivity increases over 200 (for Li^+^/Mg^2+^) and over 500 (for K^+^/Mg^2+^) even in the presence of competing ions.

## 2 Results and Discussion

### 2.1 Multilayered PIL‐CN/AAO Membranes for Monovalent/Divalent Ion Separation

The composite membranes were prepared in two steps. In the first step, CN was deposited onto an AAO support via CVD using melamine as a precursor (Figure 1a). AAO was selected as a substrate due to its thermal stability for the CVD deposition process and its well‐defined cylindrical nanochannels perpendicular to the membrane surface. The CVD process formed a continuous CN layer along the surface and within the nanochannels of AAO, resulting in a CN/AAO interim membrane. In the second step, a poly(ionic liquid) (PIL) layer was photografted from the CN/AAO via UV‐induced photopolymerization of 1‐allyl‐3‐methylimidazolium chloride (AmimCl), forming the final PIL‐CN/AAO composite membrane (Figure 1b). In this process, the CN layer acts as a photoinitiator, generating charge carriers that trigger the polymerization of the IL monomer at the membrane surface and within the nanochannels [37, 38]. Here, three increasing thicknesses of the PIL layer are achieved via increasing the AmimCl concentration during the photopolymerization. A gradual change in membrane appearance is observed across different preparation stages (Figure 1c), from pristine AAO to CN/AAO and finally to PIL‐CN/AAO.

**FIGURE 1:**
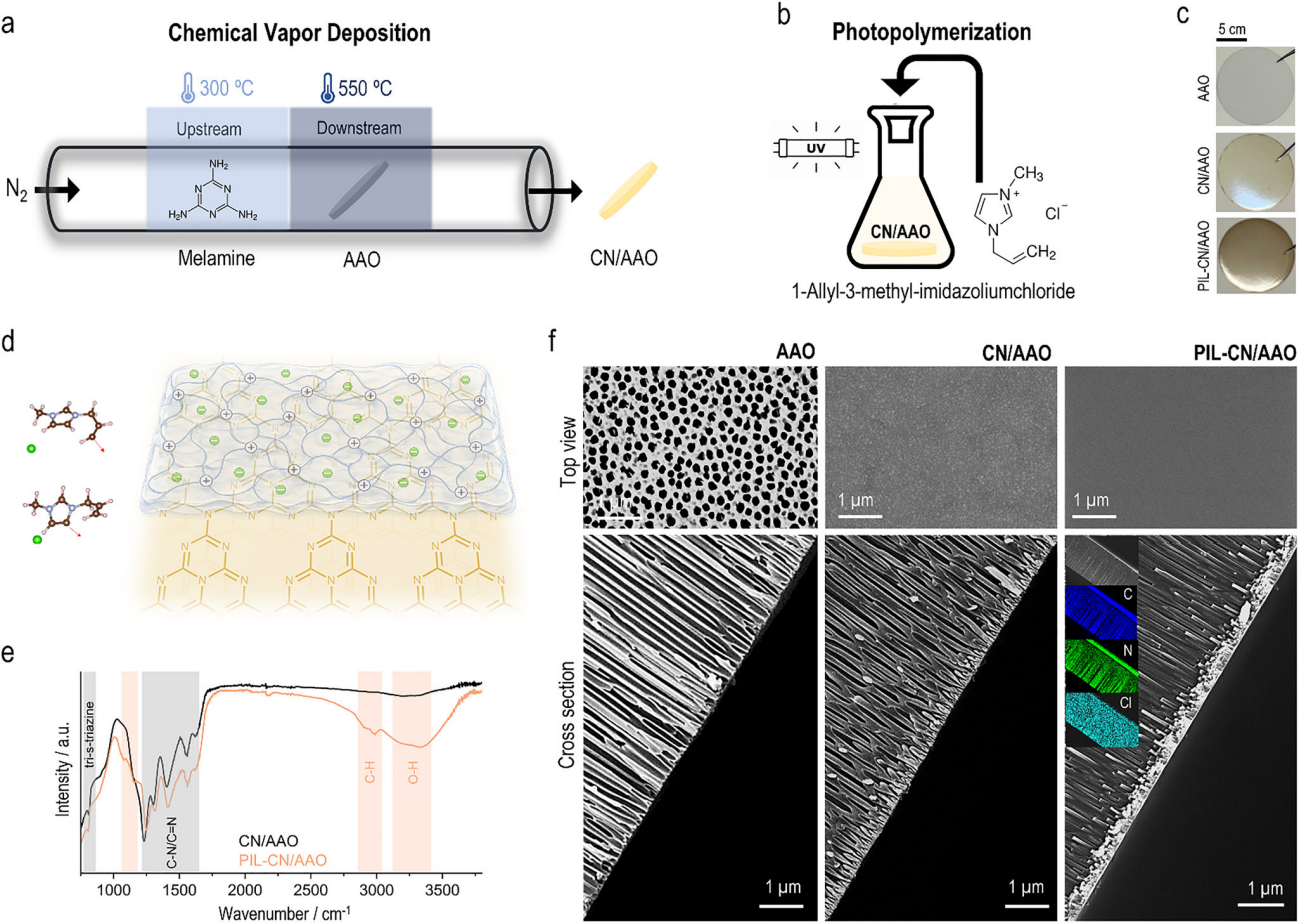
(a) Schematic illustration of the two‐step preparation process. In the first step, CN is deposited onto the AAO via CVD. (b) In the second step, the CN/AAO substrate is subjected to photopolymerization in the presence of 1‐allyl‐3‐methylimidazolium chloride monomer under UV light to form a PIL layer on the surface and nanochannels. (c) Optical images of pristine AAO, CN/AAO, and PIL‐CN/AAO membranes showing increased color contrast after each modification step. (d) Schematic representation of the proposed structure. (e) FTIR spectra of CN/AAO and PIL‐CN/AAO membranes, confirming successful incorporation of the PIL layer while retaining the characteristic CN framework. (f) SEM top view and cross‐sectional images of the membranes, showing morphological evolution from open channels in AAO to partially filled channels in CN/AAO and further constriction in PIL‐CN/AAO. The inset in PIL‐CN/AAO shows EDX elemental mapping confirming the presence of carbon (blue), nitrogen (green), and chlorine (cyan).

The calculated negative adsorption energies confirm thermodynamically favorable surface anchoring on CN, supporting the proposed surface‐confined polymerization mechanism leading to the formation of the PIL layer (Figure 1d and Table S1). Fourier‐transform infrared (FTIR) spectroscopy confirms the features of the membrane (Figure 1e). The CN/AAO membrane shows characteristic CN bands, with a tri‐s‐triazine heterocycle at 810 cm^−1^ and C─N/C═N stretching bands between 1240 and 1625 cm^−1^ [34, 39]. In contrast, the PIL‐CN/AAO membrane exhibits additional bands attributed to the PIL layer, including peaks near ∼1050 and ∼1160 cm^−1^ corresponding to imidazolium ring deformation and N─H vibrations, respectively, bands between ∼2900 and ∼2980 cm^−1^ associated with C─H stretching, and a broad feature between ∼3170 and ∼3350 cm^−1^ arising from O─H vibrations related to water adsorption [40, 41, 42].

Scanning electron microscopy (SEM) confirms the structural evolution of the membranes in both top‐view and cross‐sectional images (Figure 1f). The pristine AAO membrane shows well‐defined, vertically aligned nanochannels with diameters in the range of 20–100 nm. After CN deposition, partial filling and coating of the nanochannels are observed, indicating successful incorporation of CN within the AAO structure (Figure S1). Cross‐sectional SEM images (Figure 1f) confirm the formation of a continuous PIL layer, with thickness increasing systematically as the monomer concentration is increased. Based on the measured PIL thickness, the membranes were designated as PIL_50nm_, PIL_150nm_, and PIL_250nm_, corresponding to approximate thicknesses of 50, 150, and 250 nm, respectively (Figure S2). In addition, nanochannels that remained partially open after CN deposition are then filled by the PIL layer, indicating additional infiltration within the channels. Elemental mapping of the PIL‐CN/AAO membrane (Figure 1f) shows intense C and N signals throughout the membrane. A brighter region near the membrane surface indicates the presence of a CN‐PIL layer, while carbon and nitrogen signals detected within the nanochannels further suggest that both CN and PIL extend along the channel pathways, as further confirmed by the Cl signal arising from the PIL.

Grazing‐incidence X‐ray diffraction (GIXRD) patterns (Figure S3) show the emergence of an additional diffraction feature after PIL incorporation, indicating changes in the near‐surface structure of the composite membrane. A broad peak appears at around 2θ ≈ 10°, corresponding to a spacing of approximately 0.88 nm. This feature is associated with short‐range ordering or average interchain spacing within the PIL layer. While CN films directly deposited on flat fused silica substrates exhibit the characteristic diffraction peak at approximately 28° (Figure S3) [43], distinct CN diffraction features are not resolved in the CN/AAO and PIL‐CN/AAO membranes due to the thin CN coating and its comparably small scattering contribution relative to the AAO substrate.

Static water contact angle measurements (Figure S4) assess the changes in surface wettability resulting from membrane modification. The hydrophilic nature of the CN‐modified surface is further confirmed. Following PIL polymerization, a moderate increase in contact angle is observed with increasing PIL thickness; however, the surface remains hydrophilic, with a contact angle of 67.8° even for the thickest PIL layer. The retained hydrophilicity of the composite membrane is advantageous for this application, as improved wettability reduces interfacial resistance and facilitates efficient water and ion transport during the separation processes [44].

### 2.2 Ion Transport Under Concentration‐Driven Natural Diffusion

The ion separation performance of the membranes was first evaluated under concentration gradient‐driven natural diffusion to monitor baseline transport behavior in the absence of external stimuli [20, 45]. In these experiments, a 0.2 M equimolar multicomponent electrolyte solution containing K^+^, Na^+^, Li^+^, Mg^2+^, and Ca^2+^ was used as the feed, while the same volume of deionized water was introduced on the permeate side. Figure 2a compares the permeation rates of cations (K^+^, Na^+^, Li^+^, Mg^2+^, and Ca^2+^) across pristine AAO, CN/AAO, and PIL‐CN/AAO membranes at increasing PIL thickness. The pristine AAO membrane exhibits high ion permeation rates with negligible selectivity, consistent with its open channel structure. After the CN thin film deposition on AAO, a decrease in permeation rates is observed for all ions, reflecting partial channel modification while showing limited selectivity toward monovalent ions, as observed previously [46, 47]. In contrast, the introduction of the PIL layer leads to a pronounced decrease in ion permeation rates, with the extent of reduction strongly dependent on PIL thickness. Notably, divalent ions (Mg^2+^ and Ca^2+^) experience a stronger rejection in transport compared to monovalent ions, indicating the emergence of monovalent‐to‐divalent selectivity. This trend becomes more pronounced as the PIL layer thickness increases (Table S2). These observations point to the fact that ion rejection follows the trend of hydrated ion diameters (Figure 2a), with K^+^ exhibiting the highest permeability and Mg^2+^ the strongest rejection, despite their dehydrated ionic radii differing significantly [48]. The observed transport behavior is consistent with Fickian diffusion, where ions largely retain their hydration shells during permeation [49]. If the 0.88 nm spacing observed by GIXRD reflects the characteristic interchain spacing of the PIL layer, its close correspondence to the hydrated diameter of Mg^2+^ may place Mg^2+^ transport near a sterically constrained regime while still permitting more efficient diffusion of smaller hydrated ions. In addition, electrostatic interactions arising from the membrane further enhance selectivity through Donnan exclusion, which preferentially suppresses divalent ion transport.

**FIGURE 2:**
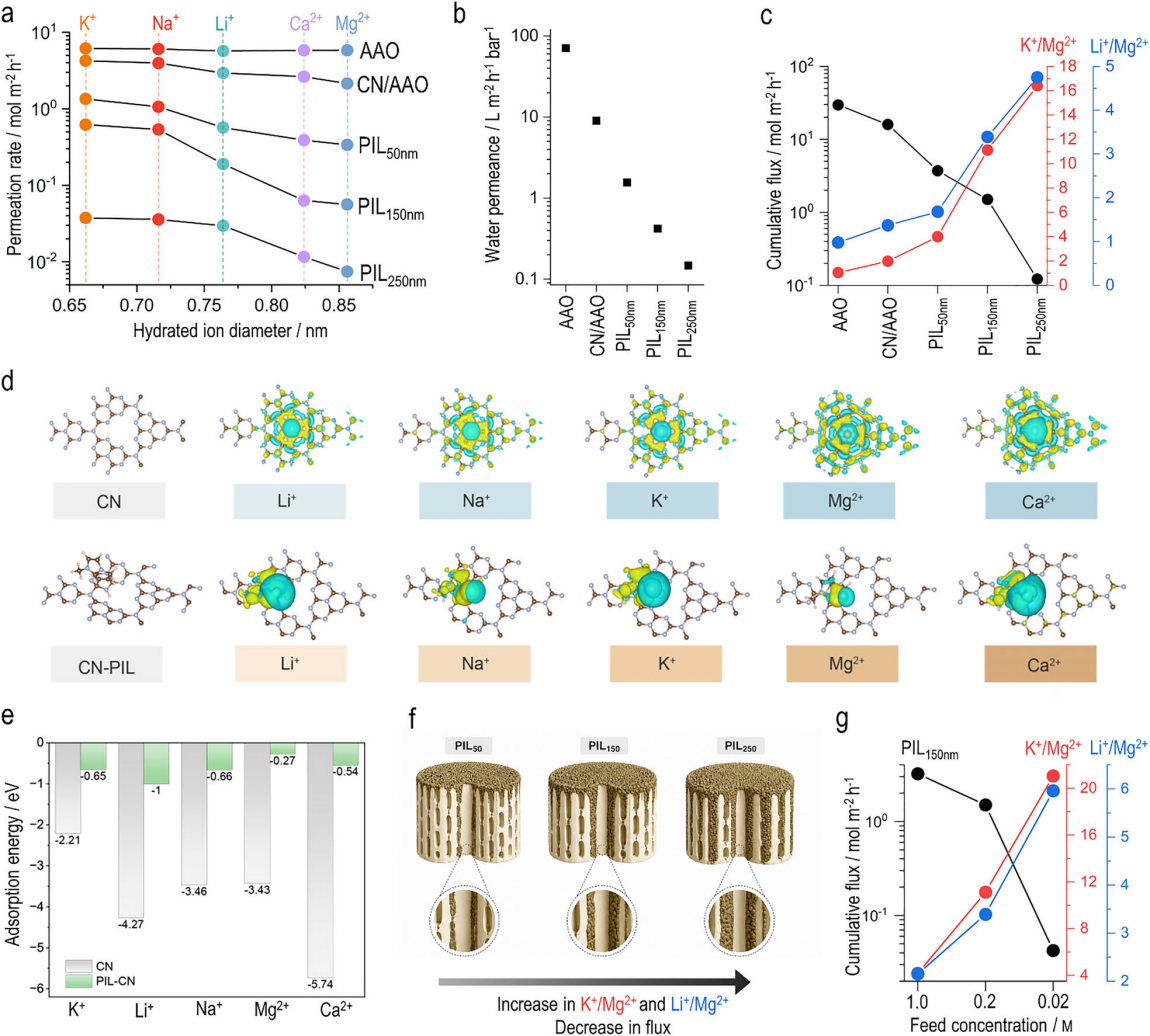
(a) Ion permeation rates plotted against hydrated ion diameter, demonstrating enhanced size‐based selectivity with increasing PIL coverage. (b) Water permeance of membranes for different membranes. (c) Cumulative ion flux (left axis, black) and selectivity ratios K^+^/Mg^2+^ (right axis, red) and Li^+^/Mg^2+^ (right axis, blue) across different membrane configurations. (d) Optimized structures of pristine CN and CN‐PIL, along with their corresponding differential charge density distributions in the presence of different ions. (e) Adsorption energies of CN‐PIL toward various ions. (f) Schematic illustration of membrane channels showing increasing pore obstruction and enhanced ion selectivity with increasing PIL thickness. (g) Effect of equimolar feed concentration on cumulative ion flux and selectivity ratios in the PIL_150nm_ membrane.

Water permeance measurements (Figure 2b) show that increasing the PIL thickness leads to a significant decrease in transmembrane water transport (Table S3). This decrease is attributed to the longer transport pathway and the higher density of polymer chains within the nanochannels, which increase hydraulic resistance and restrict the available free volume for water diffusion. Figure 2c summarizes the cumulative ion flux and selectivity ratios (K^+^/Mg^2+^ and Li^+^/Mg^2+^). Increasing PIL thickness results in a systematic decrease in overall ion flux, accompanied by a significant increase in selectivity. This behavior points to a trade‐off between selectivity and permeability that must be considered when moving toward larger‐scale operation.

Based on the optimized interfacial structures proposed by DFT calculations, the interactions of different cations with pristine CN and the CN‐PIL interface were subsequently investigated (Table S1). The charge density maps (Figure 2d) reveal substantial charge redistribution upon ion adsorption. For pristine CN, divalent ions induce stronger charge accumulation at the electron‐rich hollow sites than monovalent ions, indicating stronger electrostatic interactions with the CN framework. However, these interactions do not directly dictate ion permeation through the membrane. The transport through the CN/AAO structure is additionally governed by hydration‐dependent mobility and diffusion through the underlying AAO nanochannels. Consequently, the stronger interaction of divalent ions with the CN surface promotes transient adsorption at the CN interface, increasing their residence time before entering the underlying AAO nanochannels and thereby contributing to their lower permeation relative to monovalent ions. This further indicates that ion transport in CN/AAO is not governed by the intrinsic porous structure of the CN framework but rather by diffusion through intergranular pathways within the CN coating and the underlying AAO nanochannels, while the CN layer primarily acts as an electrostatic modifier of the transport environment.

The introduction of the PIL layer substantially modifies the local electrostatic environment and alters ion‐surface interactions. This effect is reflected in the calculated adsorption energies (Figure 2e), which reveal a decrease in the affinity of divalent ions toward the CN‐PIL interface. While pristine CN exhibits strong adsorption toward all investigated cations, particularly Ca^2+^ and Mg^2+^, the CN‐PIL structure weakens these interactions considerably. Among the monovalent ions, Li^+^ exhibits the strongest adsorption affinity toward CN‐PIL (−1.0 eV), followed by Na^+^ (−0.66 eV) and K^+^ (−0.65 eV), whereas Mg^2+^ exhibits the weakest adsorption energy (−0.27 eV). The enhanced interaction of Li^+^ is attributed to its high charge density, which promotes stronger polarization of the CN‐PIL interface. The calculated adsorption energies describe the thermodynamic partitioning of ions at the membrane interface rather than their transport through the membrane. Ion permeation is a multistep kinetic process involving adsorption, diffusion through confined nanochannels, and subsequent desorption. Consequently, stronger adsorption does not necessarily lead to higher permeability; instead, excessively strong ion‐surface interactions can increase the residence time of ions at the interface and hinder their transport across the membrane.

Among the investigated membranes, PIL_150nm_ provided the balanced compromise between ion flux and monovalent‐divalent selectivity. Based on this consideration, the PIL_150nm_ configuration was used for further investigation (Figure 2f). Figure 2g shows the relationship between cumulative ion flux and selectivity with respect to feed concentration. Increasing the feed concentration leads to a significant increase in ion flux, driven by the larger concentration gradient across the membrane, which enhances diffusive transport according to Fick's law. However, at higher concentrations, increased ionic strength perturbs electrostatic interactions within the membrane and reduces the effectiveness of Donnan exclusion, resulting in lower monovalent‐to‐divalent selectivity (Table S4) [50].

Decreasing the feed concentration reduces the overall ion flux but enhances monovalent‐to‐divalent selectivity. Under lower ionic strength conditions, reduced ion‐ion correlations enhance electrostatic interactions between the cations and the positively charged PIL layer, strengthening Donnan exclusion and amplifying differences in hydration and mobility between monovalent and divalent ions [51]. This effect is particularly observed for the K^+^/Mg^2+^ pair, as K^+^ possesses a relatively small hydrated diameter and low hydration energy, allowing it to diffuse more readily through the membrane, while strongly hydrated Mg^2+^ ions experience increased resistance. As a result, the selectivity ratios for K^+^/Mg^2+^ and Li^+^/Mg^2+^ increase to approximately 21 and 6, respectively. These results demonstrate that, in addition to membrane thickness and structural design, feed concentration is a critical parameter for tuning the balance between ion flux and selectivity under diffusion‐driven conditions.

While the main permeation measurements reported above were obtained after 24 h of membrane operation, additional experiments were conducted at different operation windows to monitor the evolution of selectivity (Figure S5 and Table S5). At 0.02 M, both K^+^/Mg^2+^ and Li^+^/Mg^2+^ increase steadily with time. This behavior reflects the suppression of Mg^2+^ transport as diffusion proceeds, while monovalent ions with higher mobility continue to permeate, resulting in enhanced monovalent‐to‐divalent selectivity under low ionic strength conditions. At a feed concentration of 0.2 M, the increase in selectivity with time is less pronounced, and after approximately 48 h both K^+^/Mg^2+^ and Li^+^/Mg^2+^ begin to decrease. Initially, the membrane maintains preferential transport of monovalent ions; however, prolonged operation promotes ion accumulation at the membrane interface, leading to enhanced electrostatic screening and concentration polarization. As a result, the effective membrane charge becomes increasingly shielded, weakening Donnan exclusion and reducing the membrane's ability to discriminate between monovalent and divalent ions. A similar trend is observed at the highest feed concentration (1 M), where selectivity remains nearly constant during the initial 24 h, followed by a slight increase and a gradual decrease at longer operation times. However, the overall selectivity remains consistently lower than at 0.2 M, reflecting the stronger electrostatic screening and more pronounced ion‐ion interactions at higher ionic strength, which further diminish the effectiveness of Donnan exclusion throughout the separation process.

### 2.3 Dynamic Selectivity Behavior Under Photo‐Assisted Electrodialysis

Following the evaluation of concentration‐driven diffusion, the separation performance of the PIL_150nm_ membrane was investigated under electrodialysis combined with visible light irradiation (*λ* = 400 nm) to probe the coupled influence of an external electric field and light absorption (Figure 3a). Upon light absorption by the CN layer, it generates surface charges, as previously shown [35], increasing the surface potential [34, 52]. This photoinduced interfacial polarization modifies the electrostatic boundary conditions within the confined PIL nanochannels, lowering the effective transport resistance. Illumination establishes a spatially resolved surface potential along the channel walls, generating an electrostatic gradient that lowers the effective migration barrier toward the permeate side. Even in the absence of an applied electric field, light illumination leads to increased permeation rates for all ions compared to natural diffusion, in particular for monovalent ions (Figure 3b,c). Due to their higher mobility and lower hydration energy, monovalent ions’ transport is significantly more favored by the light‐induced surface potential changes, whereas strongly hydrated divalent ions experience a comparatively smaller enhancement. Consequently, K^+^/Mg^2+^ and Li^+^/Mg^2+^ selectivities increase to approximately 20 and 8, respectively (Tables S6 and S7).

**FIGURE 3:**
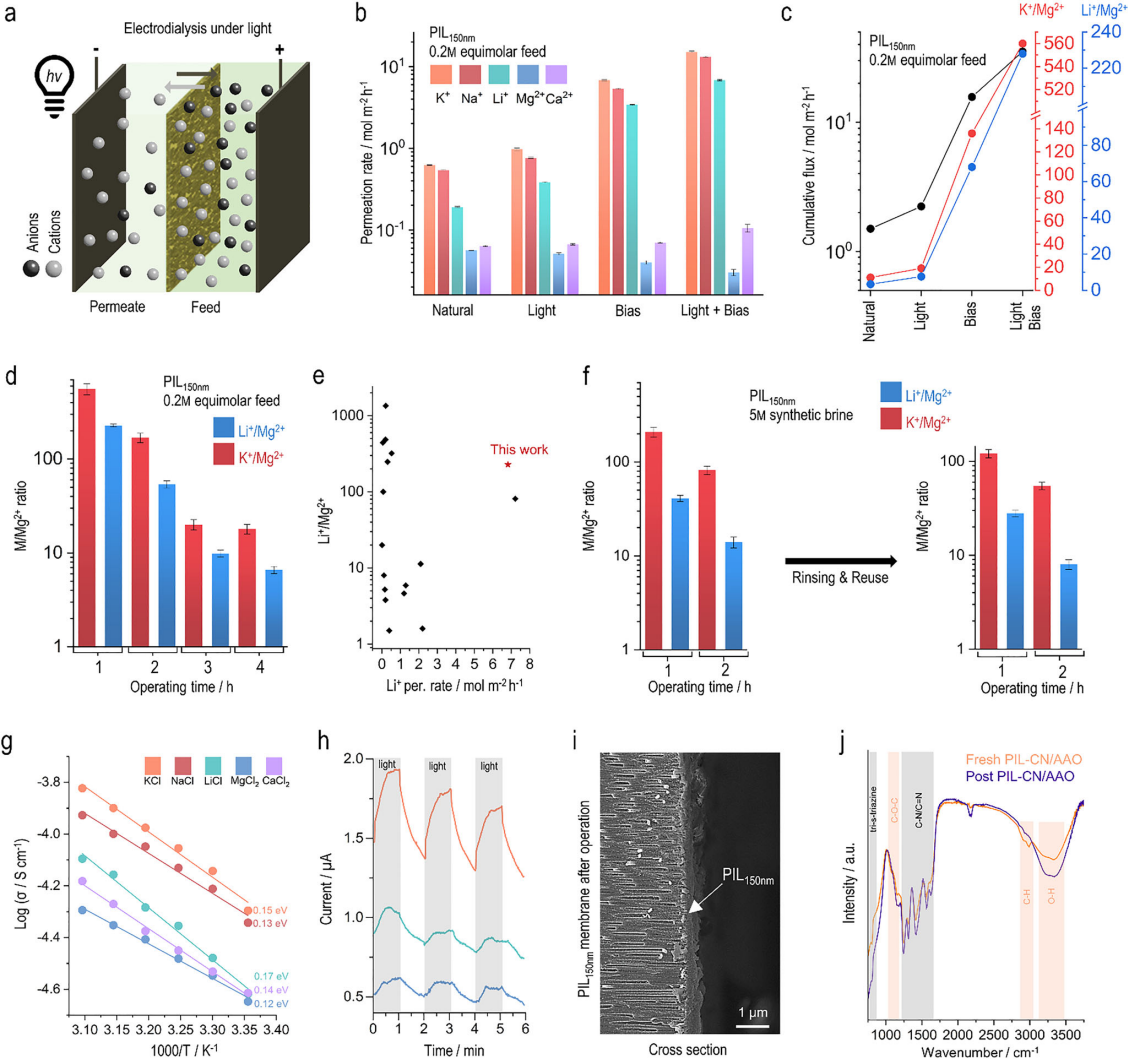
(a) Schematic of light‐assisted electrodialysis using the PIL_150nm_ membrane. (b) Ion permeation rates under natural diffusion, light, bias, and combined light + bias. (c) Cumulative ion flux and K^+^/Mg^2+^ and Li^+^/Mg^2+^ selectivity under different operating modes. (d) Time‐dependent Li^+^/Mg^2+^ and K^+^/Mg^2+^ selectivity under light + bias. (e) Literature comparison of Li^+^ permeation rates and Li^+^/Mg^2+^ selectivity for representative membranes operating under electrodialysis conditions (1 h operation, Table S10). The data point labeled “This work” corresponds to the PIL_150nm_‐CN/AAO membrane evaluated after 1 h of operation. (f) Selectivity in synthetic brine over consecutive separation cycles with rinsing and reuse. (g) Arrhenius plots of ionic conductivity and corresponding activation energies using PIL_150nm_‐CN/AAO as the membrane. (h) Light‐switching chronoamperometry under different electrolytes. (i) Cross‐sectional SEM image of the membrane after operation. (j) FTIR spectra of fresh and post‐operation PIL‐CN/AAO membranes.

The coupled action of photocharging and external bias establishes a nonequilibrium electrostatic gradient within the confined ionic domains, thereby programming ion migration pathways under operation [53]. The application of an electrical bias alone significantly increases both the cumulative ion flux and monovalent/divalent selectivity compared to diffusion‐driven transport, reflecting the enhanced electrophoretic migration of ions through the membrane. Under these conditions, ion transport is no longer governed solely by diffusion but is driven by the externally applied electric field, which couples directly to ion charge and mobility. Consequently, ions with lower hydration energy and smaller effective hydrated size exhibit higher electrophoretic mobility, whereas strongly hydrated divalent ions experience greater frictional resistance and migrate more slowly. Upon simultaneous light irradiation and electrical bias, the ion permeation rate increases by more than one order of magnitude, while the transport difference between monovalent and divalent ions is further amplified. As a result, the K^+^/Mg^2+^ and Li^+^/Mg^2+^ selectivities increase from 136 to 68 under bias alone to approximately 560 and 228, respectively, demonstrating a clear synergistic effect between photoexcitation and the applied electric field (Figure 3b,c, Tables S6 and S7).

The time‐dependent selectivity was evaluated at different operation times (Table S8). As shown in Figure 3d, exceptionally high selectivity appears during the initial stage, reflecting an out‐of‐equilibrium transport regime where electrostatic discrimination and mobility differences dominate. These results reveal an out‐of‐equilibrium transport regime in which photoinduced surface charging, coupled with an external electric bias, amplifies electrostatic discrimination within confined ionic domains, a mechanism that we define as photoelectrostatic gating.

Over time, a gradual decrease in selectivity is observed for both cation pairs, followed by stabilization after approximately 4 h. Under these conditions, the K^+^/Mg^2+^ and Li^+^/Mg^2+^ selectivities converge to values of around 18 and 7, respectively, which are comparable to those obtained under natural diffusion without light irradiation or applied bias. This observation indicates that, although photo‐assisted electrodialysis significantly enhances selectivity at early stages, the system does not maintain this amplified selectivity during prolonged operation. Nevertheless, under natural diffusion conditions, similar selectivity values are reached only after much longer periods (∼24 h), demonstrating that the combined application of light and electric bias substantially accelerates the separation process. The subsequent decrease in selectivity under photo‐assisted electrodialysis can be attributed to ion accumulation at or near the membrane surface and within the transport pathways, which reduces the effective electrostatic contrast experienced by incoming ions. As ion concentration increases within the nanochannels, electrostatic exclusion weakens, and the Donnan effect becomes less pronounced.

To benchmark the separation performance of the PIL_150nm_‐CN/AAO membrane, the Li^+^ permeation rate and Li^+^/Mg^2+^ selectivities were compared with representative electrodialysis‐based membrane systems reported in the literature. For a consistent comparison, the values obtained after 1 h of operation were used, as this time interval is commonly reported in such studies to evaluate early‐stage transport performance before significant concentration polarization and ion accumulation occur (Table S10). As shown in Figure 3e, the PIL‐CN/AAO membrane exhibits a Li^+^ permeation rate of 6.82 mol m^−2^ h^−1^ together with a Li^+^/Mg^2+^ selectivity of 228, placing it among the best‐performing lab‐scale membranes reported so far.

To further evaluate the membrane under high‐salinity conditions, separation experiments were performed using a 5 M synthetic brine with a cation ratio of Na^+^:K^+^:Li^+^:Mg^2+^:Ca^2+^ = 96:14:3.6:32:1 (Mg/Li ≈ 8.9). This composition represents a more demanding separation environment than the equimolar electrolyte, combining high ionic strength, a large excess of Mg^2+^ relative to Li^+^, and simultaneous competition among five cation species. At such high ionic strength, strong Debye screening substantially suppresses long‐range electrostatic interactions within the charged nanochannels, further challenging electrostatic ion discrimination. Despite these conditions, the membrane retained pronounced monovalent/divalent selectivity, reaching K^+^/Mg^2+^ and Li^+^/Mg^2+^ values of 208 and 41 after 1 h, respectively (Figure 3f and Table S9). Although lower than in the equimolar electrolyte, these values demonstrate robust selectivity under highly concentrated, Mg‐rich conditions. Following prolonged operation, the membrane was rinsed with Milli‐Q water, re‐equilibrated, and reused with fresh brine. The K^+^/Mg^2+^ and Li^+^/Mg^2+^ selectivities recovered to 121 and 28 after 1 h, respectively, substantially exceeding the values measured at the end of the preceding separation cycle (Figure 3f and Table S9). This partial recovery indicates that the time‐dependent performance loss is at least partly reversible and suggests that periodic washing and re‐equilibration could provide a simple strategy to mitigate selectivity decay and extend membrane operation under concentrated Mg‐rich conditions.

The electrochemical transport behavior of the PIL_150nm_ membrane was further investigated by electrochemical impedance spectroscopy (EIS), current‐voltage (*I*‐*V*) measurements, and light‐switching chronoamperometry. For these electrochemical characterizations, individual electrolyte solutions were used to probe the intrinsic transport kinetics of each ion independently and avoid competitive transport effects arising in multicomponent electrolytes. In particular, the EIS measurements were conducted to determine the ion‐specific ionic conductivity and the corresponding activation energies through Arrhenius analysis (Figures 3g and S6). Membrane impedance was recorded over a range of temperatures to monitor the intrinsic transport behavior of each ion. The Nyquist plots reveal a clear dependence of resistance on cation, with the impedance trend following K^+^ < Na^+^ < Li^+^ < Ca^2+^ < Mg^2+^, consistent with the permeation behavior observed under diffusion‐ and field‐driven conditions. MgCl_2_ and CaCl_2_ exhibit significantly larger semicircles than monovalent ions, indicating higher transport resistance arising from thermodynamically stronger hydration shells, enhanced electrostatic interactions with the PIL layer, and increased interfacial polarization (Figure S6).

Although the overall conductivity trend follows the same order observed in the permeation experiments, notable differences are observed in the activation energies. Among the investigated electrolytes, the membrane measured in LiCl exhibits the highest activation energy (∼0.17 eV), indicating a stronger temperature dependence of ionic conductivity. This behavior could be due to the strong hydration of Li^+^, which requires additional thermal energy to facilitate ion mobility through the membrane. Surprisingly, the lowest activation energy is observed for the Mg^2+^ system (∼0.12 eV) (Figure 3g). Despite its strong hydration and lower absolute mobility, Mg^2+^ transport appears less sensitive to temperature. Even at higher temperatures, Mg^2+^ retains its large hydration shell, close to the characteristic transport dimension of the membrane. This size constraint imposes a persistent steric limitation that cannot be fully overcome by thermal activation.

The electrochemical transport behavior of the PIL_150nm_ membrane was further examined using *I*‐*V* measurements and light‐switching chronoamperometry. The *I*‐*V* curves (Figure S7) show a nonlinear increase in current with applied voltage for all electrolytes, confirming field‐driven ion transport through the membrane [54]. Higher currents are observed for electrolytes containing ions with lower hydration energy, reflecting their higher electrophoretic mobility and lower resistance during transport. Periodic illumination reversibly switches the nanoionic transport state, consistent with nonequilibrium relaxation of confined ionic domains and interfacial concentration polarization (Figure 3h). The fully reversible current response confirms that the enhancement originates from photoactivation rather than irreversible structural or electrochemical changes. The current response is most pronounced in the KCl electrolyte (Δ*i*_on‐off_ = 0.505 µA), indicating that weakly hydrated ions respond more strongly to photoinduced charge generation at the CN interlayer. In comparison, LiCl exhibits an intermediate response (Δ*i*_on‐off_ = 0.140 µA), while MgCl_2_ shows the lowest current difference due to its strong hydration shell (Δ*i*_on‐off_ = 0.115 µA), reduced electrophoretic mobility, and increased electrostatic interaction with the positively charged membrane.

To directly probe the photoelectrostatic gating mechanism, the transmembrane open‐circuit voltage (OCV) was monitored under alternating light/dark conditions under symmetric 0.2 M equimolar multicomponent electrolyte. Illumination induced an immediate and reproducible negative shift in the OCV, which relaxed toward its initial value after switching off the light over consecutive cycles (Figure S8). This reversible potential response indicates photoinduced charge redistribution within the CN‐PIL architecture, demonstrating that light dynamically modulates the electrostatic potential across the membrane. Such modulation supports the electrostatic gating mechanism through which the ionic transport environment is modified under visible light. In contrast, pristine AAO showed no measurable light‐dependent potential response (Figure S8), confirming that the effect originates from the photoactive hybrid membrane. The thermal contribution to the ion separation has been ruled out since the temperature remained practically constant (±0.1°C).

To further assess structural stability, the membrane was characterized after prolonged operation in synthetic brine. Post‐operation SEM shows that the PIL layer remains intact without apparent delamination or morphological degradation (Figure 3i). Cross‐sectional EDX mapping confirms preservation of the membrane composition, while residual Na, K, Mg, Ca, and Cl signals indicate partial ion retention after rinsing (Figure S9). Consistently, FTIR spectra retain the characteristic bands of the PIL and CN framework after operation (Figure 3j). The moderate decrease in C─H stretching intensity may arise from changes in the local polymer environment associated with residual ion adsorption and prolonged exposure to the concentrated electrolyte, consistent with the residual ionic species detected by EDX. Importantly, no new bands indicative of substantial chemical or photochemical degradation are observed.

Furthermore, another control experiments performed with a CN/AAO membrane without the PIL layer in KCl show a slight light‐induced current stabilization; however, this response is negligible compared to that of the PIL‐CN/AAO membrane, confirming that the photo‐modulated transport behavior mainly arises from the coupled CN‐PIL interaction rather than the CN layer itself (Figure S10).

To simulate the effect of increased photogenerated electron–hole pair density—or equivalently, elevated light intensity—the internal electric field was enhanced by using DFT simulations (Figure 4a). The binding energy of the CN layer toward Li^+^ exhibits exceptional stability, with fluctuations confined to a narrow range from −1.860 to −1.880 eV. This minimal variation confirms that the electrostatic perturbation preserves the integrity of the strong coordination bond between Li^+^ and the CN surface, ensuring Li^+^ partitioning into and favorable interaction with the membrane's nanochannels. In contrast, under increasing simulated photoexcitation (0.01– 0.1 V Å^−1^), the binding energy of Mg^2+^ systematically weakens, as evidenced by the gradual shift toward less negative values (from −0.253 to −0.240 eV), indicating a measurable reduction in adsorption strength. The projected density of states (PDOS) was evaluated to determine the orbital interactions of CN‐PIL toward Li^+^ and Mg^2+^ ions. The positive energy shifts of the unoccupied Li 2*s* (Figure 4b) and 2p (Figure S11) states under an applied electric field of 0.1 V Å^−1^ further confirm the enhanced orbital‐specific interaction between Li^+^ ions and the nitrogen sites in CN‐PIL. The Mg^2+^‐induced features in the electronic structure exhibit no discernible changes upon application of the electric field. MD simulations further capture the dynamic configurations and structural evolution of the system in aqueous solution (Figures 4d and S12). Radial distribution function (RDF) analysis of ion‐PIL interactions shows that the Mg^2+^‐N(PIL) pair exhibits a weak peak at a larger distance (∼3.75 Å) compared to the pronounced one of Li^+^‐N(PIL) interaction (∼3.15 Å) (Figure 4c), pointing once more to less favorable transport across the membrane. This shift indicates that Mg^2+^ sits further away from the imidazolium nitrogen due to its lower polarizability and higher charge density, resulting in an increased electrostatic repulsion from the imidazolium. In contrast, Li^+^ with its lower charge density shows more favorable and stronger interactions within the confined nanochannels, supporting our interpretation of the photoelectrostatic gating effect in these PIL‐CN membranes (Figure 4e).

**FIGURE 4:**
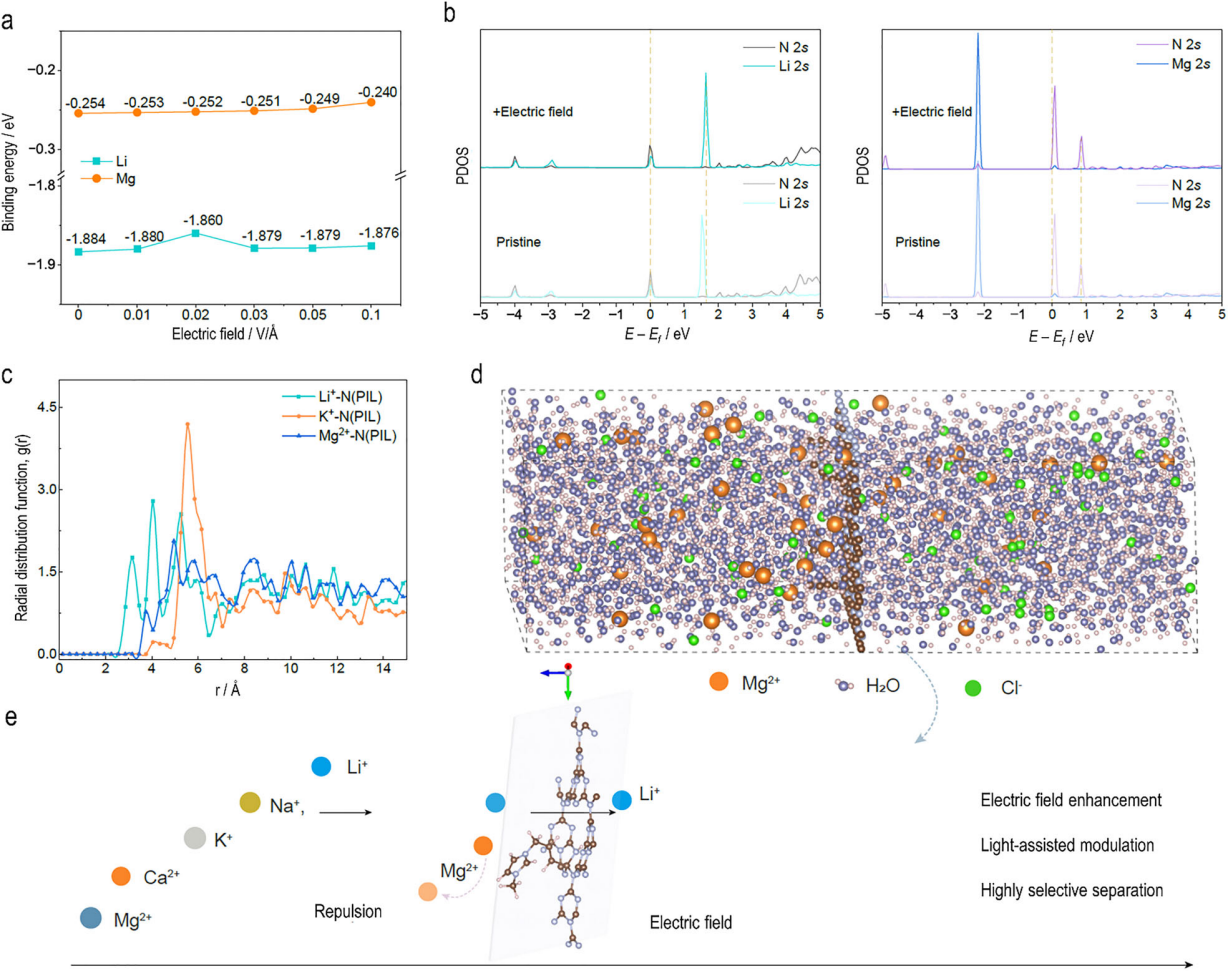
(a) Binding energies of CN‐PIL for Li^+^ and Mg^2+^ ions under varying electric fields. (b) PDOS comparison of N 2*s* and Li 2*s*/Mg 2*s* orbitals in the absence and presence of an applied electric field of 0.1 V Å^−1^. (c) Radial distribution function plots stemming from MD simulation of M(Li^+^, K^+^, and Mg^2+^)‐N (PIL) bonds. (d) MD simulations of MgCl_2_ solution within the membrane of the single‐layer CN‐PIL system. (e) Schematic illustration of the CN‐PIL‐mediated selective ion transport mechanism.

From a practical perspective, the present architecture provides a proof‐of‐concept for photoelectrostatically gated ion separation, while further development will require adaptation of both the membrane support and illumination configuration. Although AAO was employed as a model substrate owing to its well‐defined nanochannel structure, the membrane architecture is not inherently restricted to this support. The vapor‐phase CN growth can be directly extended to thermally stable porous substrates, including carbonaceous materials [33, 55], while transferable free‐standing CN films [47, 56] and lower‐temperature synthesis routes [57, 58] may enable integration with flexible polymeric supports. At the module level, external illumination of densely packed membranes would be limited by light attenuation; however, integrated light sources, internally illuminated modules, optical fiber‐assisted light delivery, or transparent flow channels could provide more effective photon distribution, as demonstrated in photocatalytic membrane systems [59, 60]. Together, these strategies provide potential routes toward translating the photoelectrostatic gating concept from the present model architecture to scalable membrane configurations.

## 3 Conclusion

The multi‐layered PIL‐CN/AAO membrane enables programmable monovalent‐divalent ion separation through electrostatic confinement within grafted poly(ionic liquid) nanochannels. Under concentration‐driven diffusion, ion transport is governed by hydrated ion size and intrinsic ion‐membrane electrostatic interactions, with consistent trends observed across different electrolyte binary systems and in complex electrolyte solutions, indicating that selectivity is primarily controlled by interfacial ion‐membrane interactions. The integration of a CN thin film as an interlayer introduces dynamic control over transport via light‐induced surface charging. Upon illumination, interfacial polarization reshapes the electrostatic energy landscape within the confined ionic domains, modulating migration barriers and transport resistance. When combined with an external electric field, this photoinduced charging establishes a nonequilibrium transport regime in which electrostatic selectivity is dynamically amplified. By coupling light and an electric field, we observed Li^+^/Mg^2+^ and K^+^/Mg^2+^ selectivities exceeding 200 and 500, respectively. At longer exposure times, relaxation toward steady‐state transport reflects interfacial ion accumulation and partial charge compensation, highlighting the nonequilibrium nature of the process. Overall, these results establish a photoelectrostatic gating mechanism in which coupled surface charging and electric bias dynamically regulate ion transport within confined nanochannels, providing a framework for stimulus‐responsive selective ion separation.

## Author Contributions

**Enis Oğuzhan Eren**: writing – original draft, conceptualization, visualization, software, formal analysis, supervision, data curation, methodology, validation, investigation, writing – review and editing. **Xuesong Xie**: writing – review and editing, software, formal analysis, data curation, methodology, investigation. **Zhi Li**: writing – review and editing, project administration, resources, investigation, funding acquisition. **Paolo Giusto**: conceptualization, funding acquisition, writing – review and editing, project administration, resources, supervision, investigation, validation.

## Conflicts of Interest

The authors declare no conflicts of interest.

## Supporting information




**Supporting File**: anie74731‐sup‐0001‐SuppMat.pdf.

## Data Availability

The data supporting this article have been included as part of the Supporting Information.
