# Supplementary material for: Hierarchical Membrane for Photoelectrostatically Gated Monovalent‐Divalent Ion Separation
Source: Angew Chem Int Ed Engl. 2026 Sep 10;65(44):e7341167. doi: 10.1002/anie.7341167 (PMC13703887; doi:10.1002/anie.7341167)
Supplement: Supplementary file 1 [file ANIE-65-e7341167-s001.pdf]

Supplementary Information

Hierarchical membrane for photoelectrostatically gated monovalent-divalent ion separation

Enis Oğuzhan Eren,[a] Xuesong Xie,[b] Zhi Li,[b] and Paolo Giusto*[a]*

[a] *Department of Colloid Chemistry, Max Planck Institute of Colloids and Interfaces, Potsdam 14476, Germany*

[b] *Department of Chemical and Materials Engineering, University of Alberta, Edmonton, Alberta T6G 1H9, Canada*

**E-mail; enis.eren@mpikg.mpg.de; paolo.giusto@mpikg.mpg.de*

Experimental Section

Deposition of carbon nitride (CN) thin films by chemical vapor deposition (CVD). CN thin films were deposited onto AAO substrates by chemical vapor deposition (CVD) following a procedure previously developed in our laboratory.[1, 2] A PlanarGROW-3S-OS CVD system (3-inch quartz tube) was used, with melamine (10 g, Sigma-Aldrich) serving as the CN precursor. The AAO substrates (Whatman alumina Anodisc filter discs ~20-100 nm pore size) were placed in the downstream heating zone, while melamine was positioned in an upstream zone to enable controlled vapor-phase delivery. After evacuation to 10 Torr, the substrate temperature was raised to 550 °C under a nitrogen flow (50 sccm). Once the substrate temperature was stabilized, melamine was heated to 300 °C at a rate of 10 °C min^{-1} and maintained for 30 min to induce CN deposition. The system was then allowed to cool naturally to room temperature.

Photopolymerization of poly(ionic liquid) layers on CN/AAO membranes. A poly(ionic liquid) (PIL) layer was formed on CN/AAO substrates via UV-induced photopolymerization of 1-allyl-3-methylimidazolium chloride (AmimCl, Sigma-Aldrich). AmimCl was dissolved in deionized water to prepare three monomer solutions with concentrations of 30, 50, and 80 wt. %, which were used to obtain PIL_{50nm}, PIL_{150nm}, and PIL_{250nm}, respectively. CN/AAO membranes were immersed in the monomer solution to ensure complete wetting and infiltration into the near-surface nanochannels. The substrates were then irradiated under UV light (λ = 365 nm, 50W) for 120 min at a fixed lamp-to-sample distance of 20 cm to initiate polymerization. After irradiation, the membranes were thoroughly rinsed with deionized water to remove residual monomer and loosely bound polymer. The resulting PIL-CN/AAO membranes were dried at vacuum oven at 60 °C before characterization and separation experiments.

Physicochemical and electrochemical characterizations. The crystallinity of the material was determined by grazing incident X-ray diffraction (GIXRD) using Rigaku SmartLab (Japan, Cu Kα, 0.154 nm). Thermo Scientific Nicolet iD7 (USA) spectrometer was used as a Fourier-transform infrared (FTIR) spectrometer. Inductively coupled plasma optical emission spectrometry (ICP-OES) was conducted with PerkinElmer Optima 8000 (USA). Scanning Electron Microscopy (SEM) imaging was performed using the Zeiss LEO 1550-Gemini (Germany) system with acceleration voltages of 3, 5, and 10 kV. An Oxford Instruments X-MAX (UK) 80 mm^2 detector was used to collect the energy-dispersive X-ray (EDX) data. Water contact angles were determined at room temperature using a DSA 10 video contact angle measurement system (Krüss, Germany), 10 s after placing a sessile water droplet on the surface. Electrochemical measurements were carried out using a Biologic MPG-2 potentiostat (France). Electrochemical impedance spectroscopy (EIS) was performed using an AC perturbation of 10 mV over a frequency range of 0.1 Hz to 20 kHz using two Pt sheet electrodes (1 cm^2). Measurements were performed at controlled temperatures of 25, 30, 35, 40, 45, and 50 °C, allowing sufficient equilibration time at each temperature before data acquisition. Single-salt electrolyte solutions (0.2M KCl, NaCl, LiCl, $CaCl_2$, and $MgCl_2$) were employed for EIS, *I-V* measurements, and light-switching chronoamperometry.

Nyquist plots were analyzed to extract the resistance associated with ionic transport. The resistance was obtained by fitting the spectra using an equivalent circuit model. Ionic conductivity (σ) was calculated using the relation:

$$\sigma = \frac{d}{R\,A} \tag{1}$$

where d is the distance between electrodes, R is the resistance, and A is the effective membrane area. Temperature-dependent conductivity values were fitted using the Arrhenius equation:

$$\sigma = \sigma_0 \exp(-\frac{E_a}{kT}) \tag{2}$$

where σ_0 is the pre-exponential factor, E_a is the activation energy for ionic transport, k is the Boltzmann constant, and T is the absolute temperature. Ionic current-voltage (I-V) measurements were carried out in a two-compartment H-cell configuration equipped with Ag/AgCl electrodes on both sides of the membrane. The applied voltage was swept between -0.5 and +0.5 V. The effective membrane area was 0.5 cm^2. 0.2M aqueous solutions of NaCl, KCl, LiCl, $CaCl_2$, and $MgCl_2$ were prepared and tested separately as individual electrolytes.

Ion permeation tests. Ion permeation experiments were conducted using an H-shaped two-compartment cell (Ossila, UK), with each compartment having a volume of 30 mL and an effective membrane area of 0.5 cm^2. The feed solutions contained equimolar amounts of NaCl, KCl, LiCl, $CaCl_2$, and $MgCl_2$, giving an Mg/Li molar ratio of 1:1. Thus, for total salt concentrations of 0.02, 0.2, and 1.0M, the concentration of each salt was 0.004, 0.04, and 0.20M, respectively. The permeate compartment was filled with deionized water. During permeation experiments, both compartments were continuously stirred using magnetic stirrers to minimize concentration polarization effects. Samples from the permeate side were collected at defined time intervals, as explained in the manuscript (i.e., 1, 2, 3, 4, 5, 24, 48, and 120 h) and analyzed by ICP-OES to quantify ion concentrations and determine permeation rates and selectivity (Eq. 3), where J denotes the ion flux, and C represents the corresponding ion concentration.

$$S_{A/B} = \frac{J_A^m / C_A^s}{J_B^m / C_B^s} \tag{3}$$

A synthetic brine containing NaCl, KCl, LiCl, $MgCl_2$, and $CaCl_2$ was prepared to evaluate membrane performance under high-salinity multicomponent conditions. The salts were mixed at a molar cation ratio of Na^+:K^+:Li^+:Mg^{2+}:Ca^{2+} = 96:14:3.6:32:1. The mixture was adjusted to a total salt concentration of 5M while maintaining this relative ionic composition. For photo-assisted electrodialysis experiments, the same H-cell configuration was used, equipped with two platinum sheet electrodes (1 cm^2 surface area) placed on each side of the membrane. A constant voltage of 0.2 V was applied across the cell. The feed compartment was filled with a 0.2M aqueous solution of NaCl, KCl, LiCl, $CaCl_2$, or $MgCl_2$, while the permeate compartment contained a 0.02M solution. Photo-assisted electrodialysis experiments were conducted under visible-light irradiation using a 50 W Kessil LED source (λ = 400 nm) positioned at a fixed lamp-to-sample distance of 30 cm. To

exclude thermal effects arising from light, the membrane temperature was continuously monitored using a thermocouple (TFA LOG200) during experiments. Water flux/permeance measurements were carried out in forward osmosis mode. The feed and draw compartments were filled with a 0.2M salt solution and a 2M sucrose solution, respectively. The osmotic pressure generated by the 2M sucrose solution was estimated to be approximately 49 bar at room temperature using the van't Hoff equation (Eq. 4), where c is the solution molarity, R is the gas constant ($0.08206\ L\cdot atm\cdot mol^{-1}\cdot K^{-1}$), and T is the absolute temperature. During the measurements, the liquids in both compartments were continuously stirred using magnetic stirrers to minimize concentration polarization. The water flux (J) was calculated according to Eq. 5.

$$\pi = c\ x\ R\ x\ T \quad (4)$$

$$J = \frac{\Delta V}{(A\ x\ \Delta t)} \quad (5)$$

Post-operation membrane characterization. For post-operation stability analysis, the PIL_{150nm}-CN/AAO membrane was subjected to two consecutive 2 h photo-electrodialysis cycles in synthetic brine, followed by approximately 5 days of natural diffusion in the same brine. After operation, the membrane was thoroughly rinsed three times with Milli-Q water, ultrasonicated around 10 min to remove residual salts and vacuum dried prior to characterization. The membrane surface and fractured cross-section were examined by SEM/EDX. FTIR spectroscopy was performed on the fresh and post-operation membranes to assess possible changes in the chemical structure.

Computational method. The density functional theory (DFT) calculation was based on first principles,[3, 4] which was conducted using VASP with a package file of 6.4.3. A triclinic simulation cell with dimensions of 14 × 14 × 30 Å was constructed for the calculation. The absorption energy and electron properties were calculated using the generalized gradient approximation (GGA) method with the Perdew-Burke-Ernzerhof (PBE) functional for the exchange-correlation term.[5] Projector-augmented-wave (PAW) type pseudopotentials are used to describe the ionic cores and the electronic structure.[6] Van der Waals interaction was taken into account at DFT-D4, and takes valence electrons into account using a plane-wave basis set with a kinetic energy cutoff of 400 eV. The SCF energy convergence criteria were established at 10^{-6} eV for electronic relaxation and 0.02 eV $Å^{-1}$ for the Hellmann-Feynman force on each atom. The Brillouin zone was sampled using a 4×4×1 k-point mesh for the ion insertions.

Molecular dynamics simulations: The systems were simulated by the classical MD method with a dimensional cell of 37 × 37 × 130 Å under periodic boundary conditions, comprising cations (Li^+, Na^+, and Mg^{2+}), Cl^- anions, a single-layer membrane of CN or CN-PIL, and water molecules. Water molecules were modeled using the rigid three-site configurations, with O-H bond lengths and H-O-H bond angles constrained via a SHAKE algorithm. First, the system was equilibrated under the NVT ensemble at 300 K for 200 ps with a time step of 1.0 fs to achieve structural stability. Following an annealing and equilibration procedure, long-term production simulations were conducted under the NPT ensemble at 300 K and 1 atm to optimize the cell.

A time step of 1 fs was employed, followed by an NPT simulation for 200 ps to equilibrate the system. Subsequently, the production simulations were conducted using the NVT procedure at 300 K over 500 ps, aimed at analyzing the structure evolution. The system temperature was maintained using a Nosé-Hoover thermostat with a damping constant of 2000 fs. The simulations were performed with a time step of 1 fs for a total duration of 500 ps. The radial distribution function (RDF) analysis was performed based on interactions between the imidazolium nitrogen (N1) atoms and metal ions with a bin step of 0.3 Å. The compositions and parameters of the supercells for the MD simulations are listed in **Table S11**.

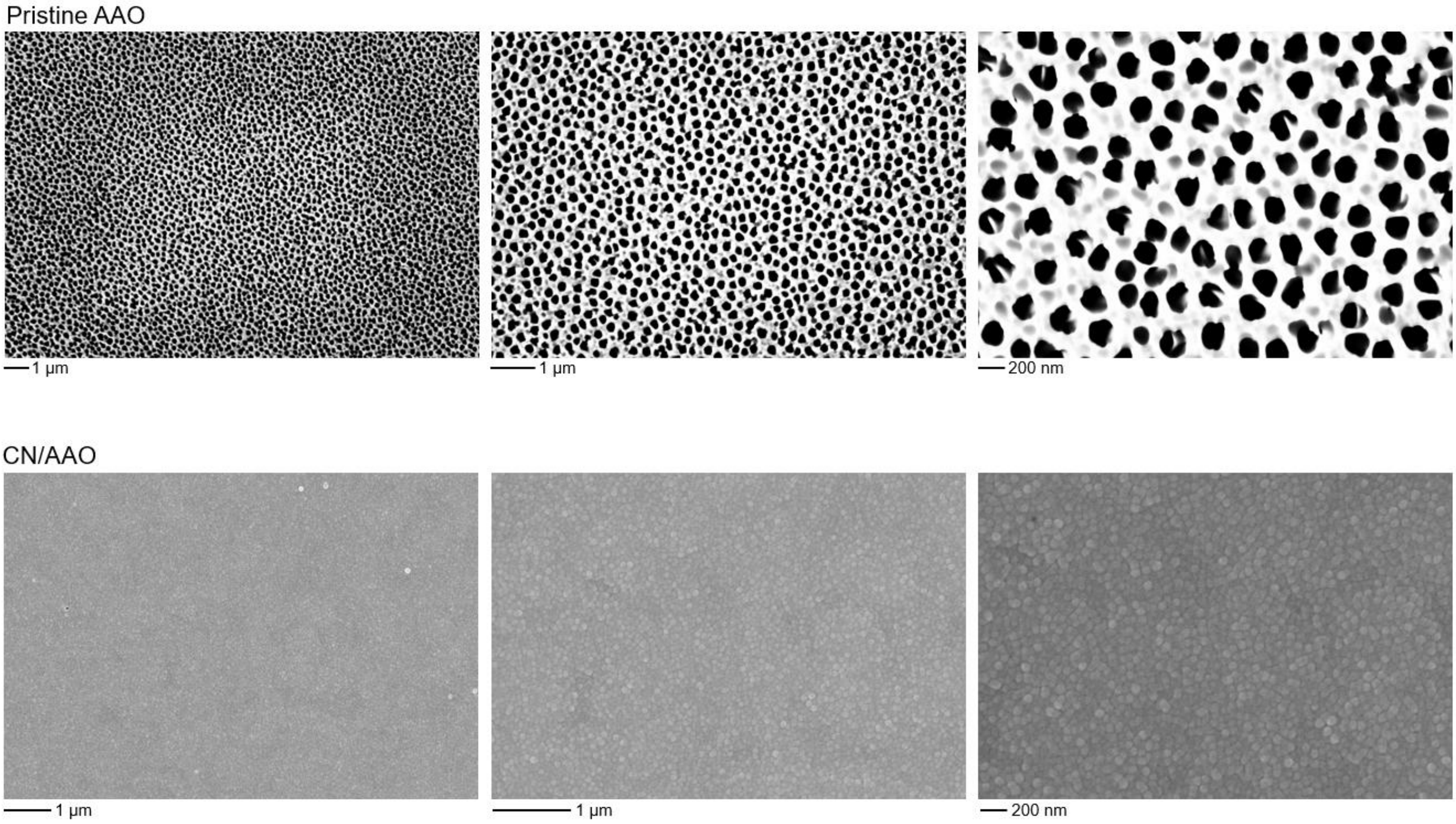

Fig. S1. SEM images of pristine AAO and CN/AAO membranes at different magnifications, showing open nanochannels in AAO and partial surface coverage after CN deposition, indicating successful modification of the nanochannel openings.

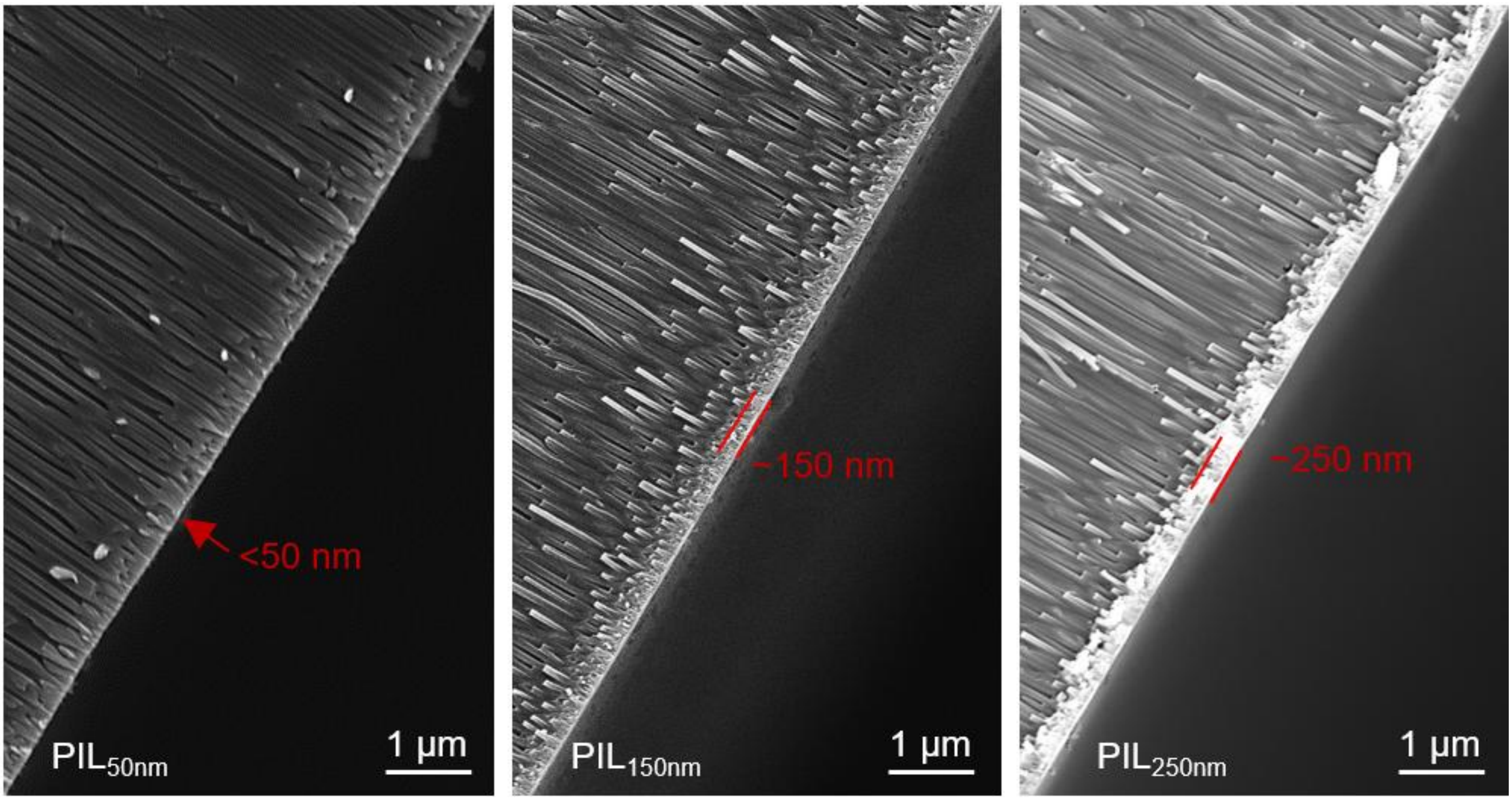

Fig. S2. Cross-sectional SEM images of PIL-CN/AAO membranes with increasing PIL thickness.

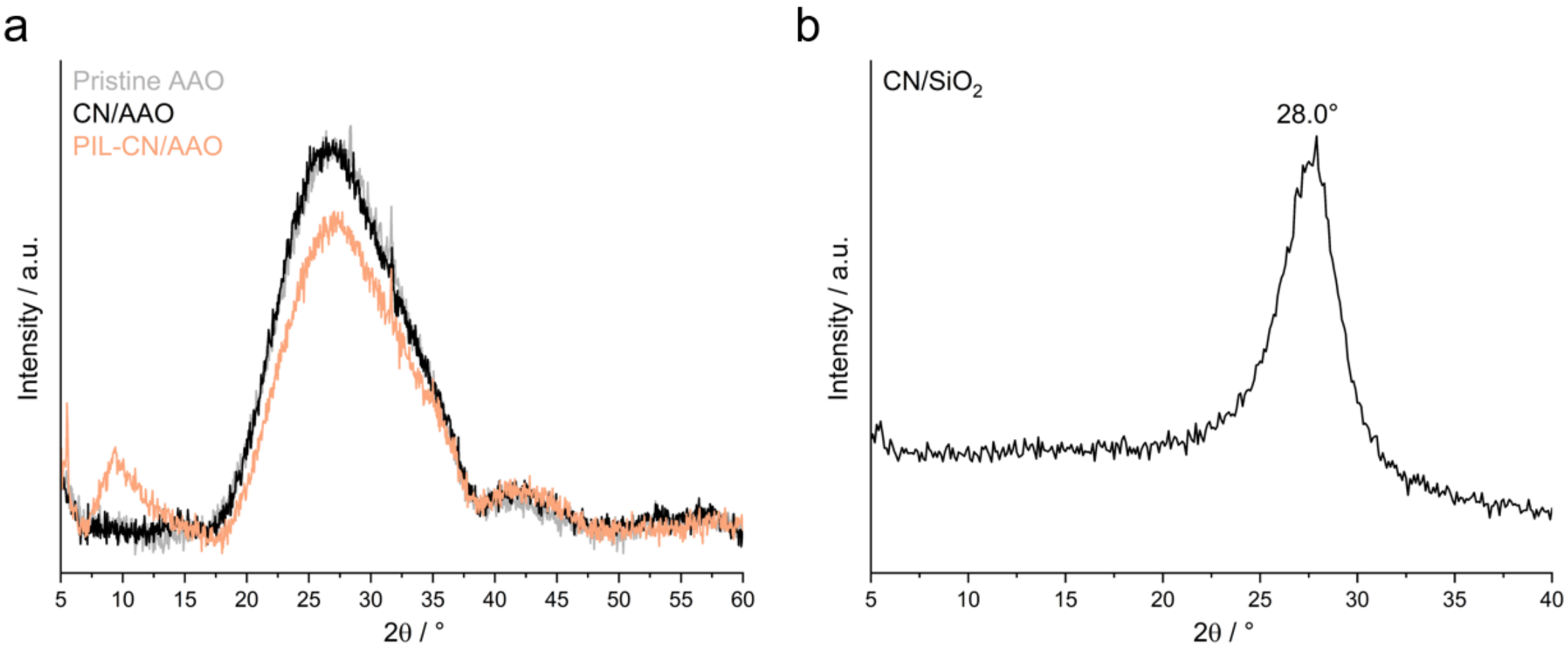

Fig. S3. (a) GIXRD patterns of pristine AAO, CN/AAO, and PIL-CN/AAO. **(b)** Reference GIXRD pattern of a CN film on SiO_2.

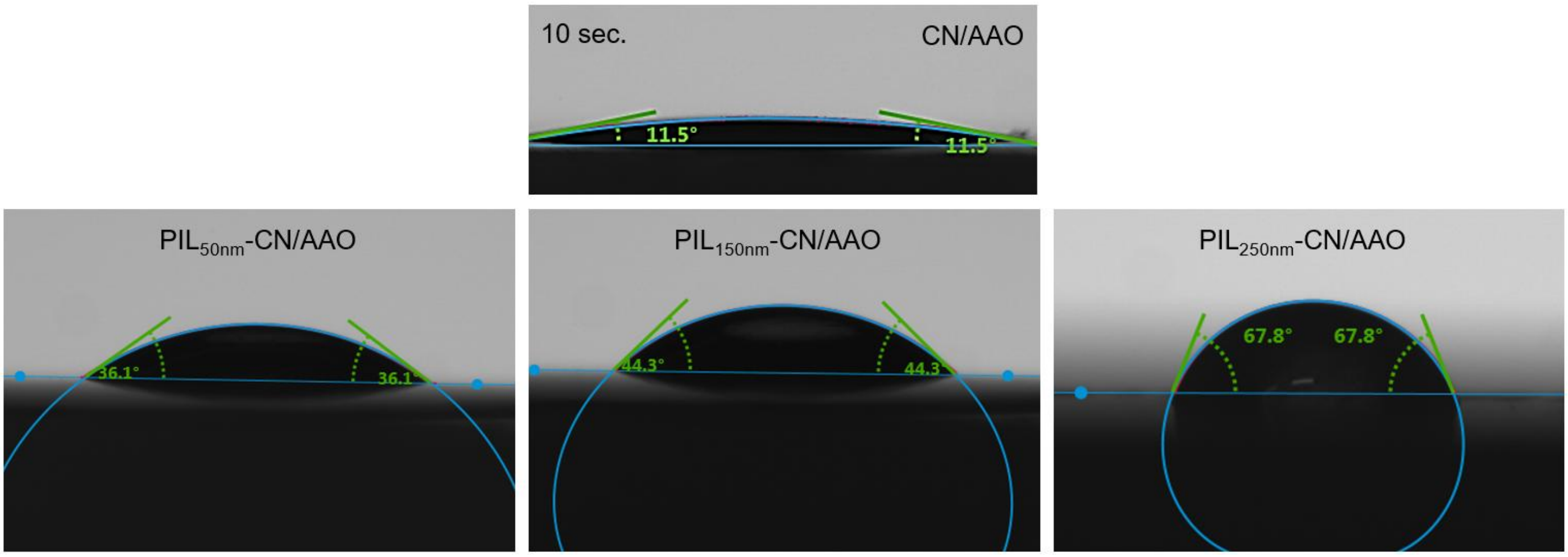

Fig. S4. Static water contact angle measurements of CN/AAO and PIL-CN/AAO membranes with increasing PIL layer thickness, showing a gradual increase in contact angle while maintaining overall hydrophilic surface characteristics.

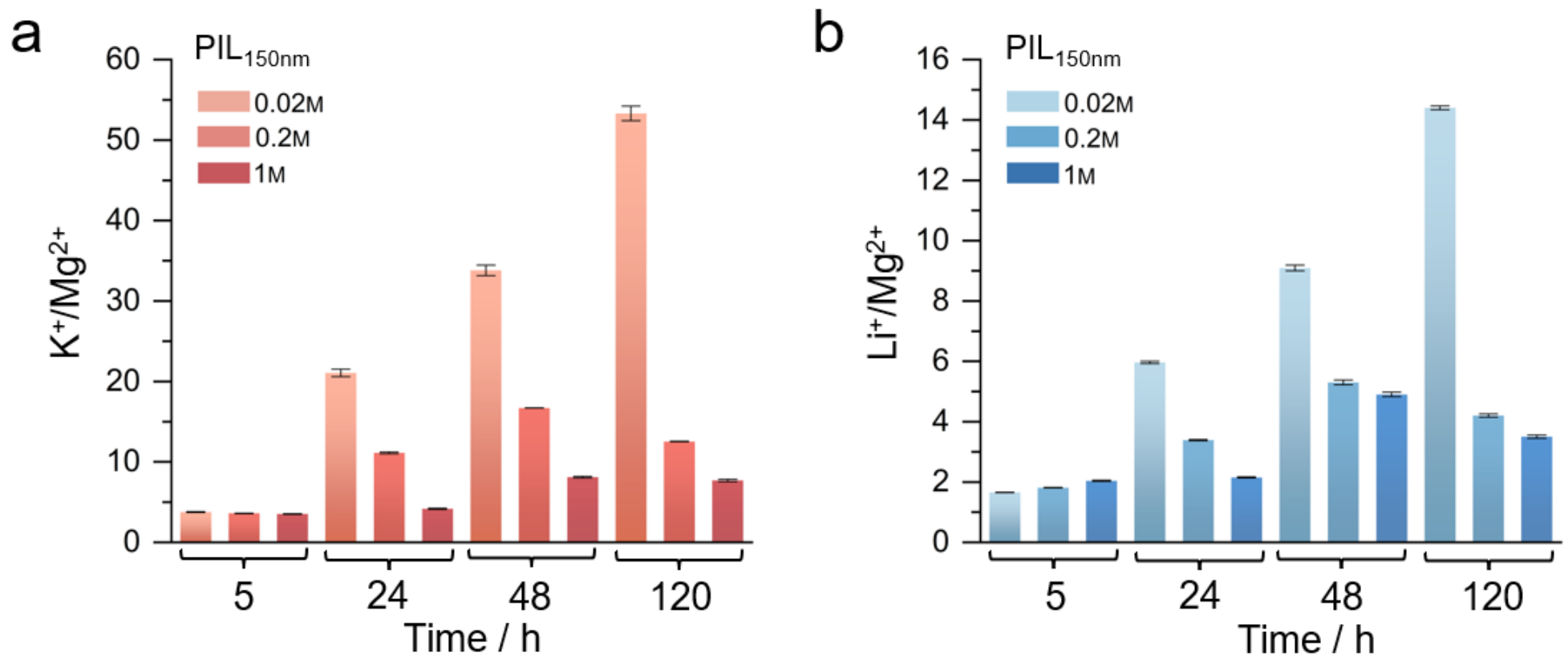

Fig. S5. Time-dependent selectivity ratios for K^+/Mg^{2+} **(a)** and Li^+/Mg^{2+} **(b)** across different feed concentrations, highlighting the evolution of selectivity at different operation times.

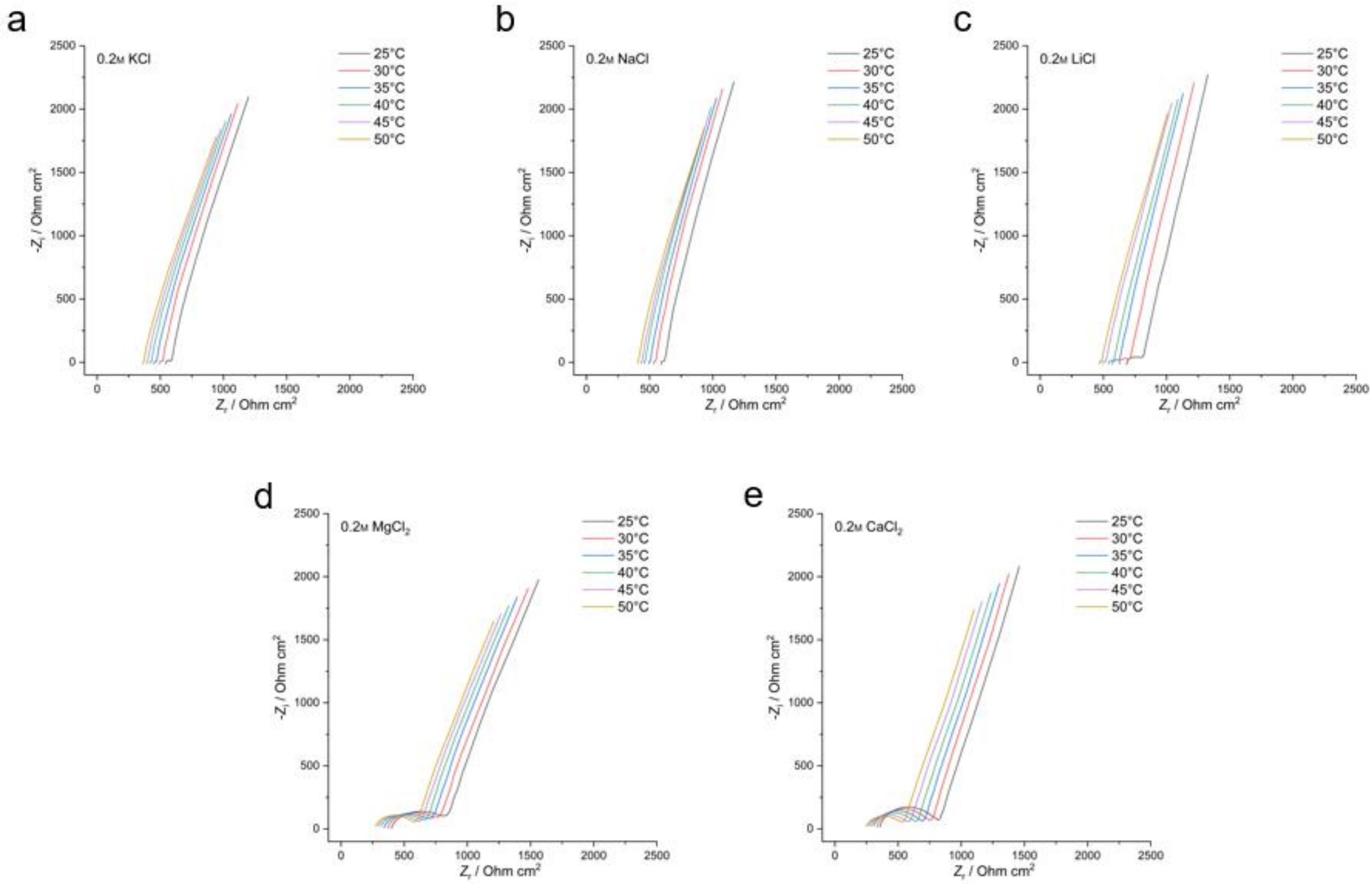

Fig. S6. Nyquist plots of the PIL_{150nm} membrane measured in 0.2M electrolytes at different temperatures (25-50 °C): **(a)** KCl, **(b)** NaCl, **(c)** LiCl, **(d)** $MgCl_2$, and **(e)** $CaCl_2$.

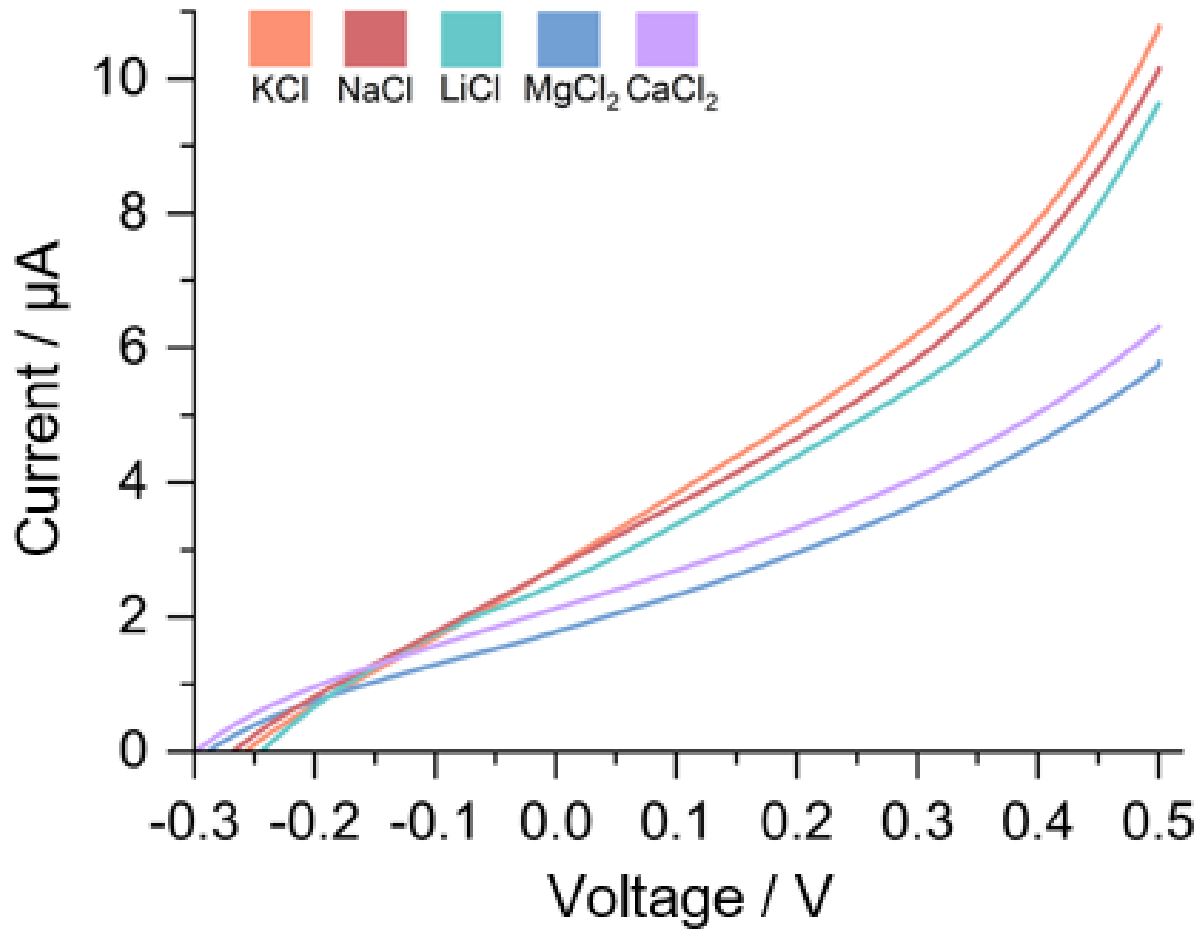

Fig. S7. *I-V* characteristics of the PIL_{150nm} membrane in different electrolytes, with monovalent cations yielding higher current.

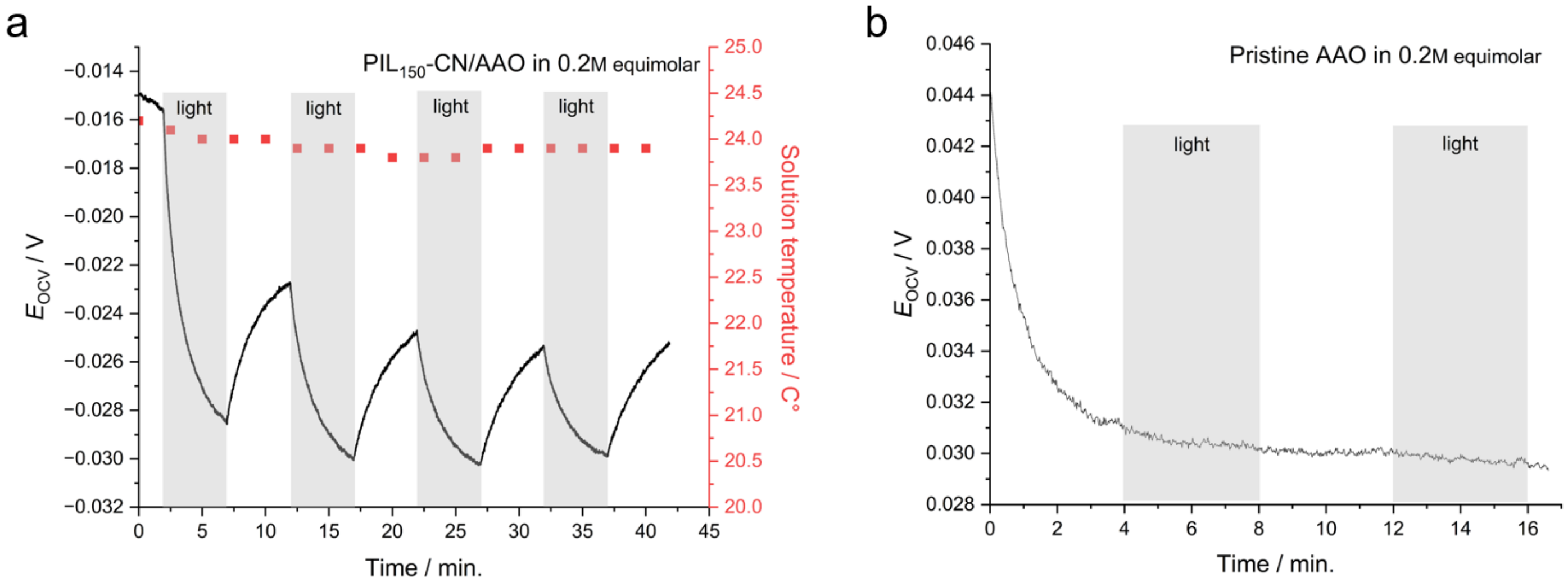

Fig. S8. Transmembrane OCV under alternating light/dark conditions in symmetric 0.2M electrolyte: **(a)** PIL-CN/AAO with simultaneous solution-temperature monitoring and **(b)** pristine AAO control. Shaded regions indicate illumination.

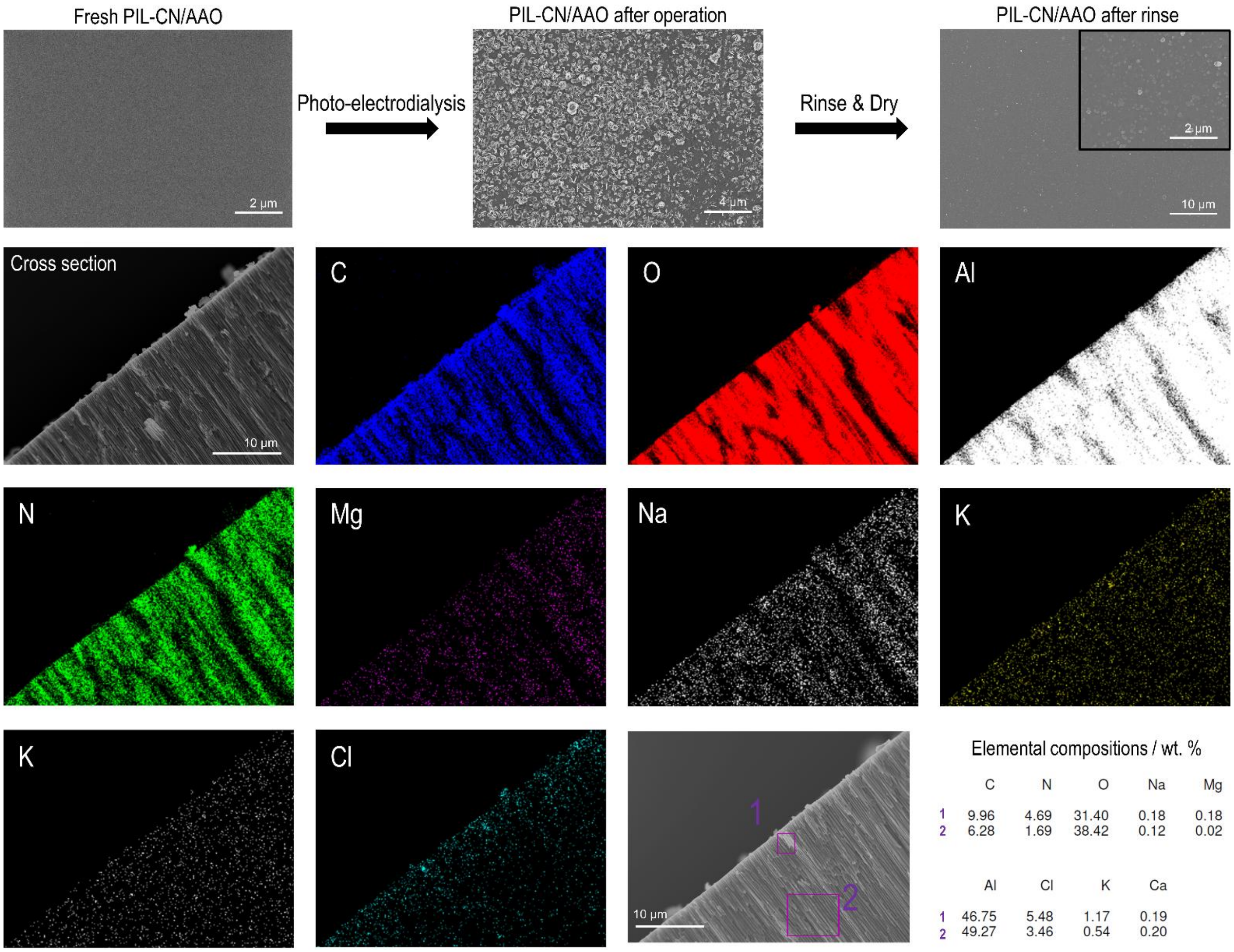

Fig. S9. SEM characterization of the PIL-CN/AAO membrane before and after photo-electrodialysis operation and following rinsing and drying. Cross-sectional SEM and corresponding EDX elemental maps show the distribution of C, N, O, Al, Mg, Na, K, and Cl after operation, rinsing, and drying. Local elemental compositions of the indicated regions are also provided.

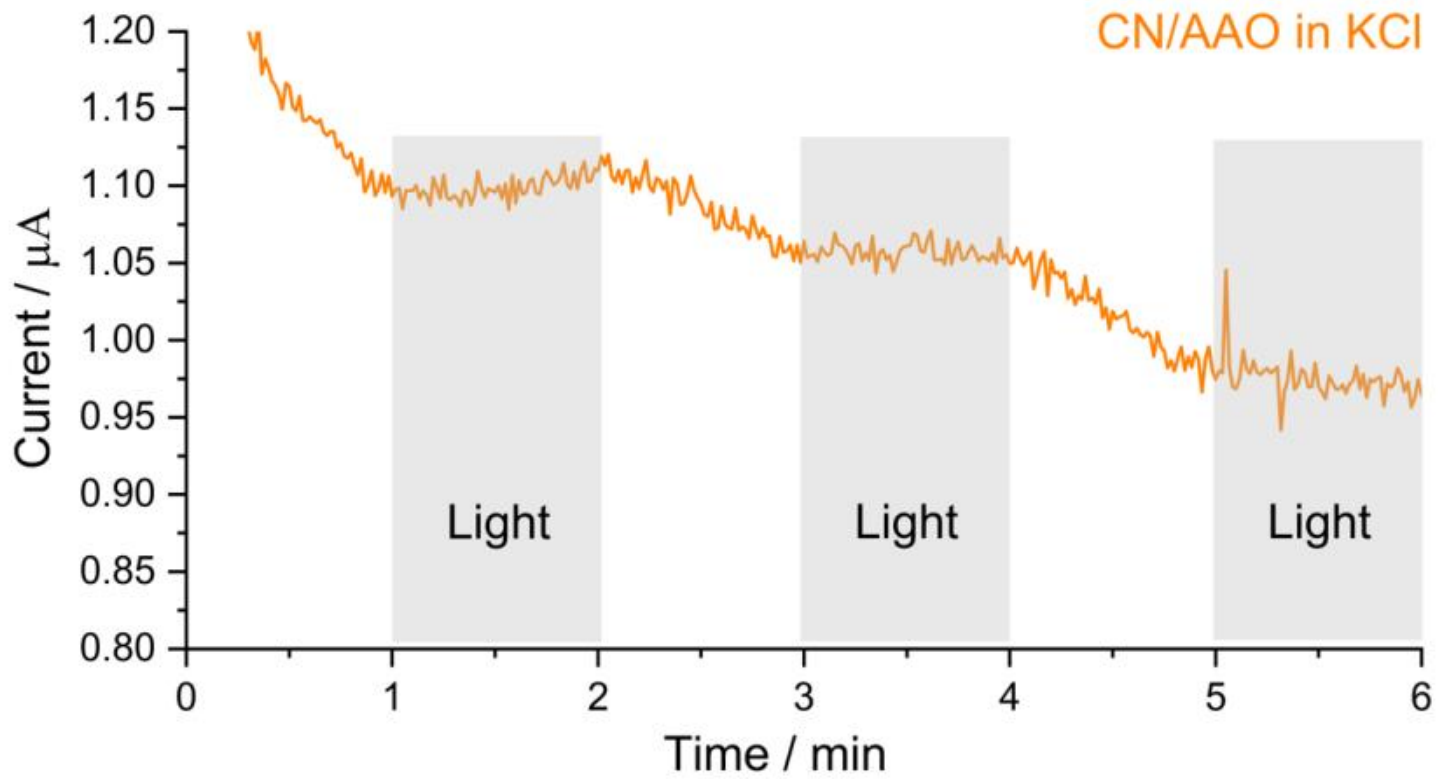

Fig. S10. Light-switching chronoamperometry of the CN/AAO membrane in KCl electrolyte. The current response is recorded under periodic illumination (gray shaded regions).

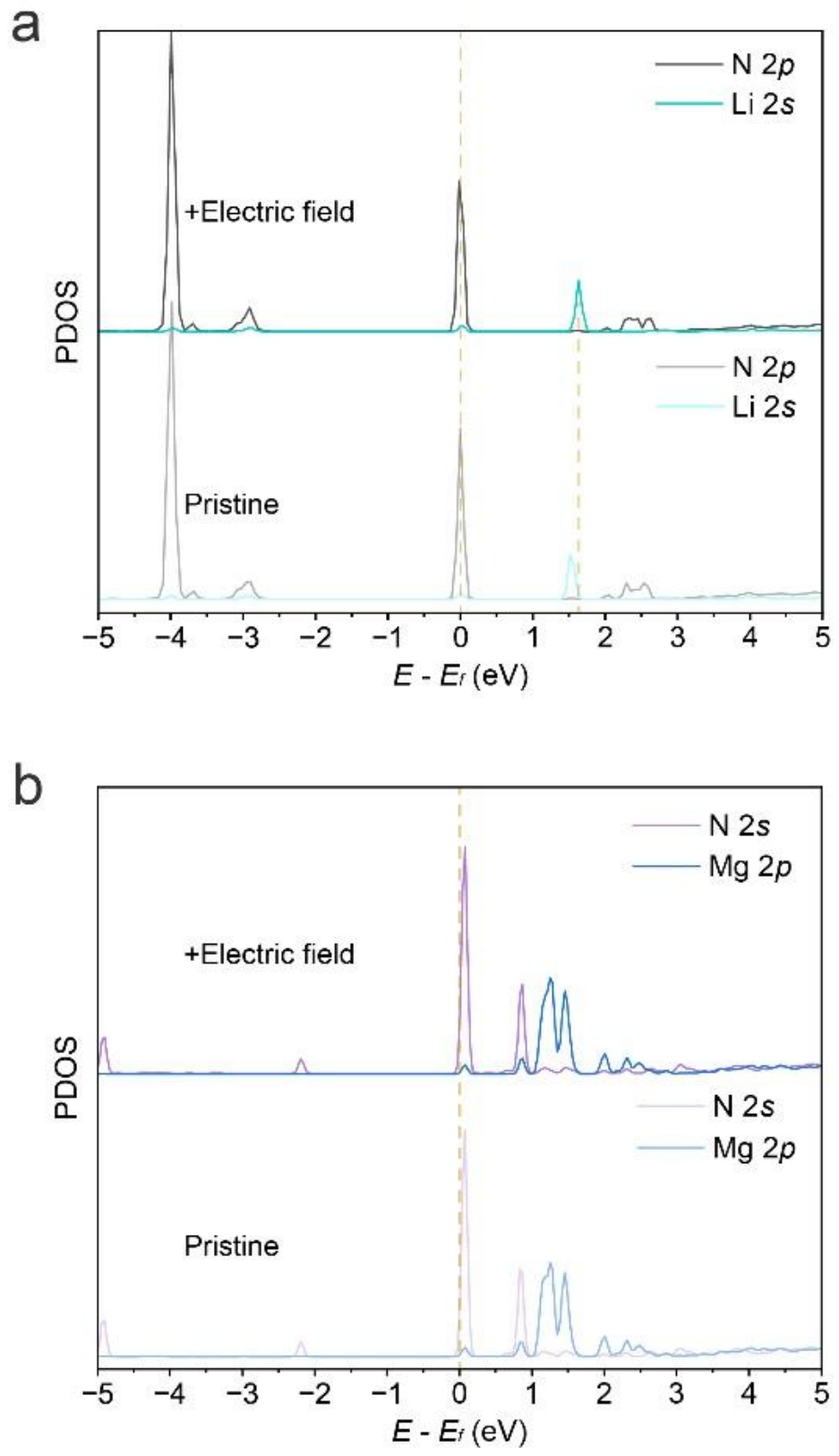

Fig. S11. PDOS comparison in the absence and presence of an applied electric field of 0.1 V $Å^{-1}$ of N 2p toward Li **(a)** and Mg **(b)** orbitals

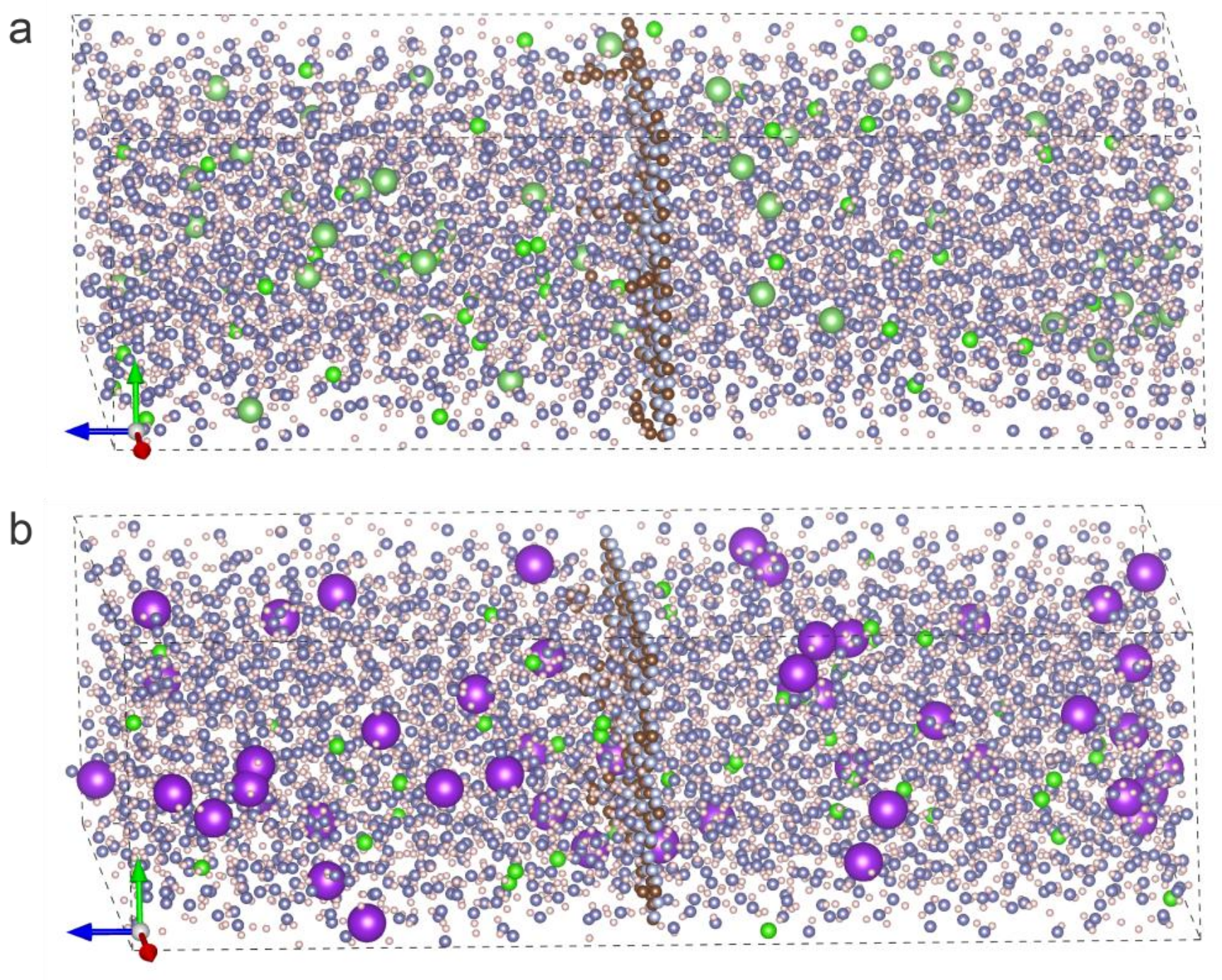

Fig. S12. MD simulations of aqueous solution within the membrane of the single-layer CN-PIL system for **(a)** LiCl, and **(b)** KCl.

Table S1. DFT-calculated adsorption energies and relative thermodynamic stability of eight possible adsorption configurations of the AMMI monomer on the CN surface.

Combination	Energy: AMMI	Energy: AMMI substrat H	Difference Energy	Thermodynamics	
**AMMI-reduceH-1**	-122.371	-115.81688	-6.55415	**Easily form**	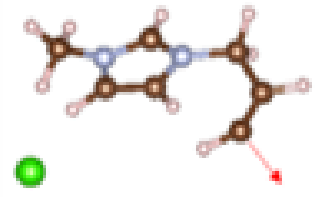
AMMI-reduceH-2	-122.371	-115.81892	-6.55211	medium	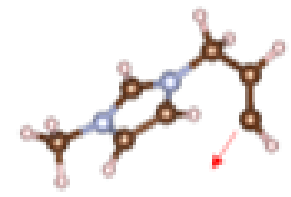
AMMI-reduceH-3	-122.371	-115.95491	-6.41612	less	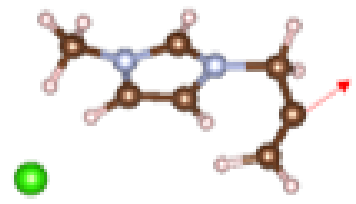
**AMMI-reduceH-4**	-122.371	-115.60552	-6.76551	**Easily form**	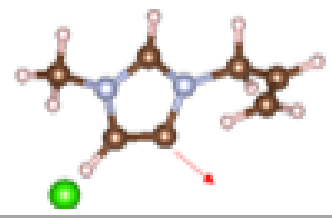
AMMI-reduceH-5	-122.371	-116.25807	-6.11296	hardly	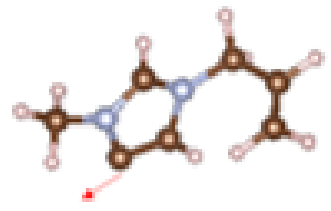
AMMI-reduceH-6	-122.371	-117.1398	-5.23123	hardly	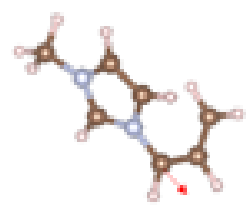
AMMI-reduceH-7	-122.371	-116.00041	-6.37062	hardly	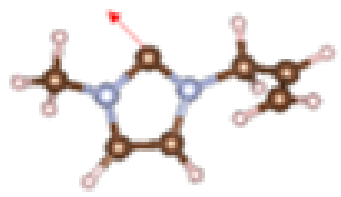
AMMI-reduceH-8	-122.371	-116.72177	-5.64926	hardly	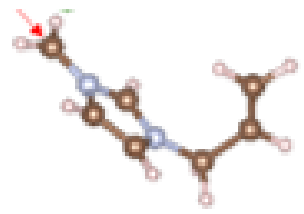

Table S2. Ion permeation rates of different membranes measured in a 0.2M equimolar multicomponent electrolyte under diffusion-driven conditions over a 24 h period.

	Permeation rate / mol m^{-2} h^{-1}				
	K^+	Na^+	Li^+	Mg^{2+}	Ca^{2+}
**AAO**	6.16±0.20	6.07±0.03	5.70±0.08	5.83±0.22	5.80±0.03
**CN/AAO**	4.26±0.16	3.97±0.07	2.95±0.05	2.15±0.13	2.64±0.1
**PIL_{50nm}**	1.35±0.02	1.06±0.01	0.57±0.01	0.34±0.01	0.39±0.01
**PIL_{150nm}**	0.622±0.01	0.538±0.01	0.19±0.005	0.056±0.0005	0.063±0.001
**PIL_{250nm}**	0.037±0.0001	0.036±0.0007	0.03±0.0002	0.0075±0.0001	0.011±0.0015

Table S3. Cumulative ion flux and water permeance of membranes measured in a 0.2M equimolar multicomponent electrolyte over a 24 h period.

	Cumulative ion flux / mol m^{-2} h^{-1}	Water permeance / L m^{-2} h^{-1} bar^{-1}
**AAO**	~29.6	71.0
**CN/AAO**	~16.0	9.0
**PIL_{50nm}**	~3.7	1.6
**PIL_{150nm}**	~1.5	0.4
**PIL_{250nm}**	~0.1	0.15

Table S4. Effect of feed concentration on cumulative ion flux and selectivity ratios for the PIL_{150nm} membrane, evaluated using equimolar multicomponent electrolytes at different concentrations over a 24 h period.

	**Cumulative ion flux / mol m^{-2} h^{-1}**	**K^+/Mg^{2+}**	**Li^+/Mg^{2+}**
**0.02M**	~0.04	21.05	5.96
**0.2M**	~1.55	11.12	3.39
**1M**	~3.2	4.17	2.15

Table S5. Selectivity ratios of the PIL_{150nm} membrane measured at different operating times in multicomponent electrolytes with varying concentrations.

	K^+/Mg^{2+}			Li^+/Mg^{2+}		
	**0.02M**	**0.2M**	**1M**	**0.02M**	**0.2M**	**1M**
**5 h**	3.76±0.04	3.6±0.04	3.52±0.03	1.65±0.007	1.81±0.01	2.0±0.02
**24 h**	21.0±0.46	11.1±0.12	4.17±0.08	5.96±0.04	3.39±0.02	2.2±0.02
**48 h**	33.8±0.65	16.7±0.02	8.1±0.11	9.1±0.1	5.3±0.08	4.9±0.07
**120 h**	53.3±0.90	12.5±0.04	7.7±0.16	14.4±0.06	4.2±0.05	3.5±0.05

Table S6. Ion permeation rates of the PIL_{150nm} membrane under different operating modes (natural diffusion, light irradiation, and combined light and electrical bias), measured using a 0.2M equimolar multicomponent electrolyte.

	**Permeation rate / mol m^{-2} h^{-1}**				
	**K^+**	**Na^+**	**Li^+**	**Mg^{2+}**	**Ca^{2+}**
**Natural diffusion**	0.622±0.01	0.538±0.01	0.19±0.005	0.056±0.0005	0.063±0.001
**Light**	0.97±0.03	0.76±0.01	0.38±0.01	0.051±0.002	0.066±0.001
**Bias**	6.82±0.1	5.33±0.09	3.4±0.04	0.04±0.002	0.07±0.001
**Light + Bias**	15.11±0.35	13.23±0.17	6.82±0.12	0.03±0.002	0.1±0.011

Table S7. Cumulative ion flux and selectivity ratios of the PIL_{150nm} membrane under natural diffusion, light irradiation, and combined light and electrical bias, measured using a 0.2M multicomponent equimolar electrolyte.

	**Cumulative ion flux / mol m^{-2} h^{-1}**	**K^+/Mg^{2+}**	**Li^+/Mg^{2+}**
**Natural diffusion**	~1.5	7.12	3.27
**Light**	~2.23	19.1	7.6
**Bias**	~15.7	136	68
**Light + Bias**	~35.3	560	228

Table S8. Time-dependent selectivity ratios of the PIL_{150nm} membrane at a total salt concentration of 0.2M under photo-assisted electrodialysis conditions.

	K^+/Mg^{2+}	Li^+/Mg^{2+}
**1 h**	560±75.3	228±7.5
**2 h**	169±19.8	54±4.9
**3 h**	20±2.54	9.9±0.8
**4 h**	18±2.1	6.6±0.6

Table S9. K^+/Mg^{2+} and Li^+/Mg^{2+} selectivity ratios of the PIL_{150nm}-CN/AAO membrane in synthetic brine during the initial and rinse-and-reuse separation cycles.

	**K^+/Mg^{2+}**	**Li^+/Mg^{2+}**
**1 h**	208±24.7	41±3.1
**2 h**	82±8.6	14±1.9
	Rinse & Reuse	
**1 h**	121±12.3	28±2.2
**2 h**	55±4.9	8±0.9

Table S10. Comparison of Li^+ permeation rates and Li^+/Mg^{2+} selectivity of reported membranes under electrodialysis conditions.

Membranes	Li^+ Permeation rate / mol m^{-2} h^{-1}	Li^+/Mg^{2+}	Operating time / h	Ref.
CSO	2.2	1.6	1	7
ENF-Q3	2.1	11.3	1	7
P-COOH	0.40	1.5	1	8
P-COOH-Q	0.19	3.8	1	8
P-COOH-QSQ	0.16	5.2	1	8
QAIPA-15	0.013	20	1	9
QAIPA-20	0.11	8	1	9
COF-EB-BD/PAN	0.05	443	1	10
CC3	0.077	100	0.5	11
(PEI/PSS)-GA	1.3	5.9	1	12
ICM	1.2	4.61	1	13
COF-EO_2/PAN	0.21	1352	3	14
UiO-67	7.22	81	3	15
UiO-66-$(SH)_2$/PET	12.2	184.6	96	16
COF-300-CH_x (1 mA cm^{-2})	0.31	248	n/a	17
COF-300-CH_x (5 mA cm^{-2})	0.53	321	n/a	17
PIM-SBI-OH-AO (2 mA cm^{-2})	0.20	486	1-2	18
PIL_{150nm}-CN/AAO	6.82	228	1	This work

Table S11. Compositions and parameters of supercells for molecular dynamics simulations.

	**LiCl**	**KCl**	**$MgCl_2$**
**K**	47	45	44
**Cl**	47	45	88
**H_2O**	2135	2059	2012
**Cell / Å**	37×37×130.3	36.9×36.9×129.4	36.7×36.7×128.7

References

1. Eren EO, Senokos E, Song Z, Yılmaz EB, Shekova I, Badamdorj B*, et al.* Conformal carbon nitride thin film inter-active interphase heterojunction with sustainable carbon enhancing sodium storage performance. *Journal of Materials Chemistry A* 2023, **11**(3)**:** 1439-1446.

2. Giusto P, Cruz D, Heil T, Arazoe H, Lova P, Aida T*, et al.* Shine Bright Like a Diamond: New Light on an Old Polymeric Semiconductor. *Advanced Materials* 2020, **32**(10)**:** e1908140.

3. Kresse G, Furthmüller J. Efficiency of ab-initio total energy calculations for metals and semiconductors using a plane-wave basis set. *Computational Materials Science* 1996, **6**(1)**:** 15-50.

4. Kresse G, Furthmüller J. Efficient iterative schemes for ab initio total-energy calculations using a plane-wave basis set. *Physical Review B* 1996, **54**(16)**:** 11169-11186.

5. Perdew JP, Burke K, Ernzerhof M. Generalized Gradient Approximation Made Simple. *Physical Review Letters* 1996, **77**(18)**:** 3865-3868.

6. Kresse G, Joubert D. From ultrasoft pseudopotentials to the projector augmented-wave method. *Physical Review B* 1999, **59**(3)**:** 1758-1775.

7. Sheng F, Hou L, Wang X, Irfan M, Shehzad MA, Wu B*, et al.* Electro-nanofiltration membranes with positively charged polyamide layer for cations separation. *Journal of Membrane Science* 2020, **594:** 117453.

8. Afsar NU, Shehzad MA, Irfan M, Emmanuel K, Sheng F, Xu T*, et al.* Cation exchange membrane integrated with cationic and anionic layers for selective ion separation via electrodialysis. *Desalination* 2019, **458:** 25-33.

9. Ul Afsar N, Ge X, Zhao Z, Hussain A, He Y, Ge L*, et al.* Zwitterion membranes for selective cation separation via electrodialysis. *Separation and Purification Technology* 2021, **254:** 117619.

10. Hou L, Xian W, Bing S, Song Y, Sun Q, Zhang L*, et al.* Understanding the Ion Transport Behavior across Nanofluidic Membranes in Response to the Charge Variations. *Advanced Functional Materials* 2021, **31**(16)**:** 2009970.

11. Xu T, Wu B, Hou L, Zhu Y, Sheng F, Zhao Z*, et al.* Highly Ion-Permselective Porous Organic Cage Membranes with Hierarchical Channels. *Journal of the American Chemical Society* 2022, **144**(23)**:** 10220-10229.

12. Zhang J, Lin Y, Zhang Y, Wu B, Cao X, Shi Z*, et al.* The role of ion-membrane interactions in fast and selective mono/multivalent ion separation with hierarchical nanochannels. *AIChE Journal* 2023, **69**(12)**:** e18232.

13. Zhang Y, Lin Y, Ying J, Zhang W, Jin Y, Matsuyama H*, et al.* Highly efficient monovalent ion transport enabled by ionic crosslinking-induced nanochannels. *AIChE Journal* 2022, **68**(11)**:** e17825.

14. Meng Q-W, Zhu X, Xian W, Wang S, Zhang Z, Zheng L*, et al.* Enhancing ion selectivity by tuning solvation abilities of covalent-organic-framework membranes. *Proceedings of the National Academy of Sciences* 2024, **121**(8)**:** e2316716121.

15. Xu R, Kang Y, Zhang W, Zhang X, Pan B. Oriented UiO-67 Metal–Organic Framework Membrane with Fast and Selective Lithium-Ion Transport. *Angewandte Chemie International Edition* 2022, **61**(3)**:** e202115443.

16. Zhao C, Feng F, Hou J, Hu J, Su Y, Liu JZ*, et al.* Unlocking Direct Lithium Extraction in Harsh Conditions through Thiol-Functionalized Metal–Organic Framework Subnanofluidic Membranes. *Journal of the American Chemical Society* 2024, **146**(20)**:** 14058-14066.

17. Meng W, Chen S, Guo Z, Gao F, Wang J, Lu J*, et al.* Three-dimensional cationic covalent organic framework membranes for rapid and selective lithium extraction from saline water. *Nature Water* 2025, **3**(2)**:** 191-200.

18. Yang D, Yang Y, Wong T, Iguodala S, Wang A, Lovell L*, et al.* Solution-processable polymer membranes with hydrophilic subnanometre pores for sustainable lithium extraction. *Nature Water* 2025, **3**(3)**:** 319-333.
